# An unsupervised dual-phase framework combining statistical separation and hybrid level set evolution for robust motion detection and segmentation in intelligent video surveillance

**DOI:** 10.1038/s41598-026-50215-9

**Published:** 2026-05-02

**Authors:** Meriem Boumehed, Aykut Fatih Güven, Abderrahmane Naoum, Amir Merahi, Belal Alshaqaqi, Khaled Guerraiche, Muneera Altayeb, Oleksandr Rubanenko

**Affiliations:** 1 Higher School of Electrical and Energetic Engineering of Oran, Department of Second Cycle, Smart Grid Development Laboratory, Oran, Algeria; 2 Faculty of Electrical Engineering, Department of Electronic, Signal and Image Laboratory, University of Science and Technology of Oran-Mohamed Boudiaf, Oran, Algeria; 3 Department of Electrical and Electronics Engineering, Yalova University, Yalova, Turkey; 4 National Higher School of Autonomous Systems Technology, Algiers, Algeria; 5 Hourani Center for Applied Scientific Research, Al-Ahliyya Amman University, Amman, Jordan; 6 Department of Power Plants and System, Vinnytsia National Technical University, Vinnytsia, 21000 Ukraine

**Keywords:** Active contours, Background subtraction, Fast independent component analysis, Hybrid level set model, Intelligent video surveillance, Motion detection, Motion segmentation, Unsupervised learning, Engineering, Mathematics and computing

## Abstract

Accurate detection and segmentation of moving objects constitute a fundamental challenge in computer vision, particularly for intelligent video surveillance systems operating under variable illumination, dynamic backgrounds, and environmental noise. This paper presents a fully unsupervised dual-phase motion analysis framework that effectively combines statistical independence modeling and geometric contour evolution to achieve high-precision motion detection and segmentation. In the first phase, an enhanced Fast Independent Component Analysis (Fast-ICA) algorithm is employed to perform statistical decomposition of video sequences, exploiting temporal independence to distinguish moving foregrounds from static backgrounds. This process generates an initial motion mask with strong robustness to illumination variation and noise artifacts. In the second phase, a hybrid level set segmentation model integrating the global Chan-Vese formulation and a locally adaptive Yezzi-based energy function refines object boundaries through an adaptive energy minimization process. A stabilization term and a self-regulating convergence criterion are further incorporated to ensure contour smoothness, numerical stability, and resilience to topological changes. Comprehensive experiments conducted on the CDNet-2014 benchmark dataset demonstrate that the proposed method achieves an average recall of 0.9613, precision of 0.9089, and F-measure of 0.9310, outperforming several state-of-the-art supervised, semi-supervised and unsupervised background subtraction algorithms. The proposed Fast-ICA–Level Set fusion framework thus provides a robust, adaptive, and computationally efficient solution for real-world intelligent surveillance and autonomous visual monitoring applications.

## Introduction

In recent years, moving object detection and segmentation have become critical tasks in computer vision, especially for video surveillance systems used in public security, traffic monitoring, and smart environments^1–6^. The fundamental objective is detecting and segmenting dynamic regions of interest in video sequences, enabling subsequent higher-level applications such as traffic surveillance, motion object tracking, and anomalous behavior identification. Despite significant progress, developing a robust, real-time, and generalizable approach remains challenging due to environmental complexities like sudden illumination changes, motion of static objects, occlusions, camera movements, and the presence of shadows or moving backgrounds. Moving object detection is a broad and well-explored field with various proposed methods. Among the most commonly used methods is background subtraction (BGS), which can be divided into three fundamental classes: threshold-based, supervised, and unsupervised methods. Threshold-based methods determine whether a pixel belongs to the background or foreground by evaluating the difference between the current frame and a reference model against a predefined threshold^7^. Supervised methods, particularly those based on deep learning have revolutionized this domain with models such as FG-BR-NET^8^, SSD^9^, R-CNN^10^, DPM^11^, YOLO^12^, FgSegNet_v2_CO^13^, neural-enhanced techniques (BSUV-Net 2.0)^14^, Motion U-Net^15^, and deep CNN-based background subtraction^16^ offering high accuracy in object detection and segmentation. However, these models typically require extensive labeled datasets and significant computational resources often limit their applicability in practice^7^, thus unsupervised BGS methods present compelling alternatives by avoiding the need for training excessive data and remaining more practical in the real world. Early approaches have often relied on background subtraction like Gaussian Mixture Model (GMM)^17^ and its adaptive variant^18^, boosted Gaussian mixture model with controlled complexity (BMOG)^19^, Sample-based background subtractor (SBBS)^20^, Visual Background Extractor (ViBe)^21^, the Self-Balanced Sensitivity Segmenter (SuBSENSE)^22^, WeSamBE^23^, WisenetMD^24^, RT-SBS^25^. Moreover, active contour methods^26^ are among the most widely used unsupervised techniques that operate by evolving a deformable contour object by internal and external forces derived from the image features. Existing active contour methods come in two main groups: parametric models (e.g., snakes) and geometric models (e.g., level-set methods). Parametric models explicitly represent the evolving curve and use differential equations to maintain a balance between contour elasticity and external attraction forces. In contrast, geometric models implicitly represent the evolving contour as the zero level set of a higher-dimensional embedding function, whose evolution is based on image features and geometric constraints. These methods are generally categorized into edge-based models^27,28^ and region-based models^29–33^. Edge-based models utilize local edge features, such as pixel gradients to direct contour evolution, however, they are highly sensitive to noise and tend to underperform in low-contrast or poorly defined boundary conditions. By incorporating region features like texture, color, and intensity statistics, region-based models can increase resilience to noise and enhance accuracy in delineating object boundaries. Significant methods of region-based level sets have been developed to optimize the renowned Mumford and Shah (MS) model^29^. Notably, Chan-Vese^30^ and Yezzi et al.^31^ introduced two influential approaches that characterize object regions via a bi-model segmentation. In these models, energy minimization is performed through gradient descent applied to the level set function. Nonetheless, their dependence on global image statistics often limits performance in scenarios involving heterogeneous or spatially varying object regions. To overcome intensity inhomogeneities, the Local Binary Fitting model introduced by Li et al.^34^, assuming local homogeneity within image regions. While it is marked a significant improvement, its reliance on local mean intensity limited its accuracy in high-noise settings. To address this, Wang et al.^35^ proposed the Local Gaussian Distribution method, which characterizes local intensity variations within each region using Gaussian distributions. Nevertheless, its segmentation performance degrades under non-Gaussian noise conditions. To overcome such challenges, researchers have incorporated non-local methods and Markov Random Field (MRF) models to better model spatial dependencies and reduce noise sensitivity^36^. Recent advances propose hybrid models^37–42^ to further address the challenges of image segmentation. By integrating global and local region-based active contour frameworks through a weighted combination, these methods retain the global model’s robustness while enhancing boundary detection accuracy through localized sensitivity.

Notable examples include the Global and Local Region Active Contour model^37^, the Laplace of Gaussian Functional (LOGF) proposed by Ding et al.^38^, and a diffusion-regularized level set model with an enhanced edge indicator function^39^, which collectively improve segmentation under noisy or weak boundary conditions. Further developments include bilateral-filter-based local region models^40^, which perform effectively in low-contrast settings, and the Global Symbolic Energy (GLSE) model by Liu et al.^41^, which introduces a novel global pressure force to mitigate sensitivity to initial contour placement.

Despite the extensive progress in motion detection and segmentation, a single technique rarely achieves optimal performance across the wide spectrum of conditions encountered in real-world surveillance scenarios. Environmental complexities such as illumination variation, background dynamics, intermittent motion, and shadow interference continue to challenge the robustness and generalization capability of existing algorithms. Consequently, hybrid frameworks have gained considerable attention, as they attempt to integrate complementary models to enhance accuracy and stability. For instance, the combination of wavelet optical flow with hybrid linear–nonlinear classifiers^42^ or the coupling of level set evolution with motion cues have demonstrated improved robustness in complex scenes. Nevertheless, these methods generally remain limited in their adaptability and computational efficiency, and few approaches have successfully achieved a fully unsupervised integration of statistical and geometric models. Classical background subtraction techniques, including GMM^17^, ViBe^21^, and SuBSENSE^22^, tend to fail under dynamic or cluttered backgrounds, while conventional level set formulations whether global (e.g., Chan–Vese) or local (e.g., Yezzi, LBF) are often sensitive to noise and intensity inhomogeneities, leading to boundary leakage or incomplete segmentation. Similarly, Independent Component Analysis (ICA) and its computationally efficient variant Fast-ICA^43,44^ offer a complementary statistical approach. By treating video sequences as linear mixtures of independent sources, ICA-based methods can separate motion components from background without requiring pre-modeled backgrounds or training data^45^. Early applications of ICA to motion detection demonstrated its potential for indoor and surveillance scenarios^46–48^, with later refinements improving robustness in complex environments^49^. Subsequent integrations with geometric active contours further showcased the synergy between statistical separation and boundary refinement^50^. Recent advances have introduced enhanced ICA formulations using independence measures such as the Hilbert-Schmidt criterion^51^ and improved adaptation mechanisms for dynamic scenes^52^. However, standalone ICA implementations often produce noisy, spatially incoherent foreground masks and struggle with complex multimodal backgrounds, requiring subsequent refinement stages to achieve practical segmentation quality^46^. These limitations highlight a critical gap: the absence of a unified unsupervised framework that combines the statistical separation capability of ICA with the geometric contour adaptability of hybrid level sets, thereby enabling robust, data-driven, and high-precision motion detection and segmentation across diverse surveillance environments. Addressing this problem defines the central research objective of the present study.

More recently, novel semi-supervised methods such as graph signal processing^53,54^ and graph neural networks^55–57^ have emerged, offering promising avenues for motion detection with reduced dependency on labeled data by leveraging spatial and temporal relationships in video data through graph-based representations.

Despite significant progress in background subtraction, ICA, active contours, and deep learning, existing methods still face limitations in handling dynamic backgrounds while maintaining accurate boundaries, efficiency, and unsupervised operation.

In practical surveillance environments, captured video frames are frequently affected by degradations such as motion blur, compression artifacts, atmospheric turbulence, and low-quality imaging conditions. These degradations can significantly impact the reliability of motion detection algorithms by distorting object boundaries and weakening motion cues, which may affect both statistical separation methods and contour-based segmentation. Recent advances in image restoration have demonstrated promising capabilities in recovering structural details prior to high-level vision tasks. For instance, MB-TaylorFormer v2^58^ introduces an efficient transformer-based architecture for motion deblurring, while DBLRNet^59^ proposes an effective deep neural network for dynamic scene deblurring. Similarly, MC-Blur^60^ addresses complex blur patterns using multi-component modeling. Although such restoration methods are not the focus of this study, their integration with motion segmentation frameworks represents a promising direction for improving robustness under degraded surveillance conditions.

In this paper, we propose Unsupervised Motion Detection via Background Subtraction and Hybrid Level Set (UMD-BHLS), a novel fully unsupervised framework that fuses statistical background modeling and geometric contour evolution. The proposed approach operates in two co-dependent stages:

1. Enhanced Fast-ICA decomposition for robust foreground–background separation^46^.
2. A novel adaptive hybrid level set formulation integrating global Chan–Vese consistency^30^ with local Yezzi-driven boundary sensitivity^31^.

The proposed UMD-BHLS framework is designed to balance computational efficiency with segmentation accuracy, making it suitable for real-world surveillance environments where both annotated data and computational resources are limited. To address these challenges, we introduce a unified motion detection framework that integrates statistical motion separation with adaptive contour-based segmentation within a single processing pipeline.

Unlike conventional background subtraction methods that rely exclusively on statistical modeling, or contour-based approaches that operate directly on raw image intensities, the proposed framework combines Fast Independent Component Analysis (Fast-ICA) for statistical motion separation with an adaptive hybrid level-set evolution for geometric refinement. This integration allows the system to first isolate statistically independent motion components and then refines object boundaries through contour evolution driven by image region statistics.

The key novelty of the proposed approach lies in the adaptive hybrid energy formulation, which introduces an entropy-guided global–local balancing mechanism together with a self-regulated stabilization strategy during contour evolution. This mechanism dynamically adjusts the influence of global and local energy terms according to scene characteristics, enabling the segmentation process to remain stable and accurate under heterogeneous background conditions such as dynamic textures, illumination variations, and intermittent object motion.

By coupling statistically robust motion separation with adaptive geometric contour refinement in a unified unsupervised framework, the proposed UMD-BHLS method improves foreground extraction accuracy while maintaining computational efficiency and eliminating the need for training data or manual parameter tuning.

The main contributions of this work are summarized as follows:

- Statistical-geometric integration: A unified unsupervised framework combines Fast-ICA motion separation with hybrid level set contour refinement, enabling robust foreground extraction through the joint exploitation of statistical independence and geometric boundary evolution.
- Fully adaptive parameter formulation: A novel level-set energy functional that integrates global consistency (Chan–Vese model) with locally adaptive boundary sensitivity (Yezzi model) through an entropy-guided global–local balancing mechanism.
- Self-regulating parameter optimization: Automatic estimation of key parameters, including energy balance coefficients, and regularization weights, using intrinsic scene statistics, thereby eliminating manual parameter tuning.
- Fully unsupervised operation without training data: Unlike many deep learning-based motion detection approaches, the proposed framework achieves robust segmentation performance without requiring labeled training data, making it well suited for practical surveillance systems with limited annotation resources.
- The remainder of this paper is organized as follows: Sect. 2 reviews two global level set techniques by Chan–Vese^30^ and Yezzi et al.^31^.

Section 3 elaborates on the mathematical implementation of the proposed UMD-BHLS approach. Section 4 presents experimental results, illustrating the effectiveness of UMD-BHLS on real-world image datasets, along with a comprehensive performance evaluation against several approaches. Finally, Sect. 5 concludes the paper with a summary of the proposed framework. 

## Background

### Chan and Vese model

Chan and Vese (CV)^30^ a region-based active-contour model for segmenting an image I(x, y) defined on a domain Ω. Assuming the object of interest comprises two distinct regions Ω _int_ and Ω _ext_ with uniform intensities $C_{{int}}^{g}$ and $C_{{ext}}^{g}$The model minimizes an energy functional that is optimized when these mean intensities closely approximate the respective regions. The global energy term $\mathop E\nolimits_{{cv}}^{g}$ is given as follows:

1 $$\,\,E_{{cv}}^{g}(C_{{\operatorname{int} }}^{g},\,C_{{ext}}^{g},\phi )=\int\limits_{\Omega } {{{\left| {{\mathbf{I}}\, - C_{{\operatorname{int} }}^{g}} \right|}^2}{H_\varepsilon }\left( \phi \right)dxdy} +\int\limits_{\Omega } {{{\left| {{\mathbf{I}}\, - C_{{ext}}^{g}} \right|}^2}\left( {1 - {H_\varepsilon }\left( \phi \right)} \right)dxdy}$$

The level set function ϕ is defined as the distance function D from point *(x*,* y)* to the contour Γ:

2 $$\phi \,(x,y)\,=\,\left\{ \begin{gathered} +\,D\left( {(x,y),\Gamma } \right)\,\,\,(x,y) \in {\Omega _{\operatorname{int} }} \hfill \\ 0\,\,\,\,\,\,\,\,\,\,\,\,\,\,\,\,\,\,\,\,\,\,\,\,\,\,\,(x,y) \in \,\,\Omega \hfill \\ - \,D\left( {(x,y),\Gamma } \right)\,\,(x,y) \in \,{\Omega _{ext}} \hfill \\ \end{gathered} \right.\,$$

${H_\varepsilon }\left( \phi \right)$ denotes the smoothed Heaviside function used the numerical computations:

3 $${H_\varepsilon }\left( \phi \right)=\,\left\{ {\begin{array}{*{20}{c}} {\,1\,\,\,\,\,\,\,\,\,\,\,\,\,\,\,\,\,\,\,\,\,\,\,\,\,\,\,\,\,\,\,\,\,\,\,\,\,\,\,\,\,\,\,\,\,\,\,\,\phi \,(x,y)\,>\,\varepsilon } \\ {0\,\,\,\,\,\,\,\,\,\,\,\,\,\,\,\,\,\,\,\,\,\,\,\,\,\,\,\,\,\,\,\,\,\,\,\,\,\,\,\,\,\,\,\,\,\,\,\,\,\,\,\,\phi \,(x,y)\,<\, - \varepsilon \,} \\ {\frac{1}{2}\left\{ {1+\frac{\phi }{\varepsilon }+\frac{1}{\pi }\sin \left( {\frac{{\pi \,\phi }}{\varepsilon }} \right)} \right\}\,\,\,\,\left| {\phi \,(x,y)} \right| \leqslant \varepsilon } \end{array}} \right.\,\,$$

The mean intensity values $C_{{int}}^{g}$ and $C_{{ext}}^{g}$are derived by differentiating the energy functional $\mathop E\nolimits_{{cv}}^{g}$ with respect to $C_{{int}}^{g}$ and $C_{{ext}}^{g}$, and setting them equal to zero:

4 $$C_{{\operatorname{int} }}^{g}(\phi )=\frac{{\int\limits_{{{\Omega _{\operatorname{int} }}}} {{\mathbf{I}}\,(x,y){H_\varepsilon }\left( \phi \right)dxdy} }}{{\int\limits_{{{\Omega _{\operatorname{int} }}}} {{H_\varepsilon }\left( \phi \right)dxdy} }}$$

5 $$C_{{ext}}^{g}(\phi )=\frac{{\int\limits_{{{\Omega _{ext}}}} {{\mathbf{I}}\,(x,y)\left( {1 - {H_\varepsilon }\left( \phi \right)} \right)\,dxdy} }}{{\int\limits_{{{\Omega _{ext}}}} {\left( {1 - {H_\varepsilon }\left( \phi \right)} \right)\,dxdy} }}$$

### Yezzi model

Yezzi et al.^31^ introduced a related strategy based on the same foundational principles of CV model, aiming to minimize a global energy function that incorporates the mean intensities $C_{{int}}^{g}$ and $C_{{ext}}^{g}$. This method seeks to minimize an energy expression (6), which reaches its minimum value when the mean intensities of the R_int_ and R_ext_ are maximally distinct. Consequently, the contour evolves to maximize the separation between these regional averages, enhancing contrast along the boundary.

6 $$\,\,E_{y}^{g}(C_{{\operatorname{int} }}^{g},\,C_{{ext}}^{g},\phi )= - \frac{1}{2}\int\limits_{\Omega } {{{\left( {C_{{\operatorname{int} }}^{g}\, - C_{{ext}}^{g}} \right)}^2}dxdy}$$

## Proposed approach

The proposed fully adaptive unsupervised motion detection and segmentation framework (UMD-BHLS) introduces an improved hybrid level set technique that self-adjusts its internal parameters based on the spatial and temporal scene statistics. The model dynamically balances global and local information, automatically adapts to image texture and illumination variability, and self-controls the evolution process through adaptive regularization and convergence assessment. The system operates in two successive phases:

- Statistical Motion Detection using an enhanced Fast Independent Component Analysis (Fast-ICA) method;
- Segmentation Refinement via the proposed adaptive hybrid level-set model.

**Fig. 1:**
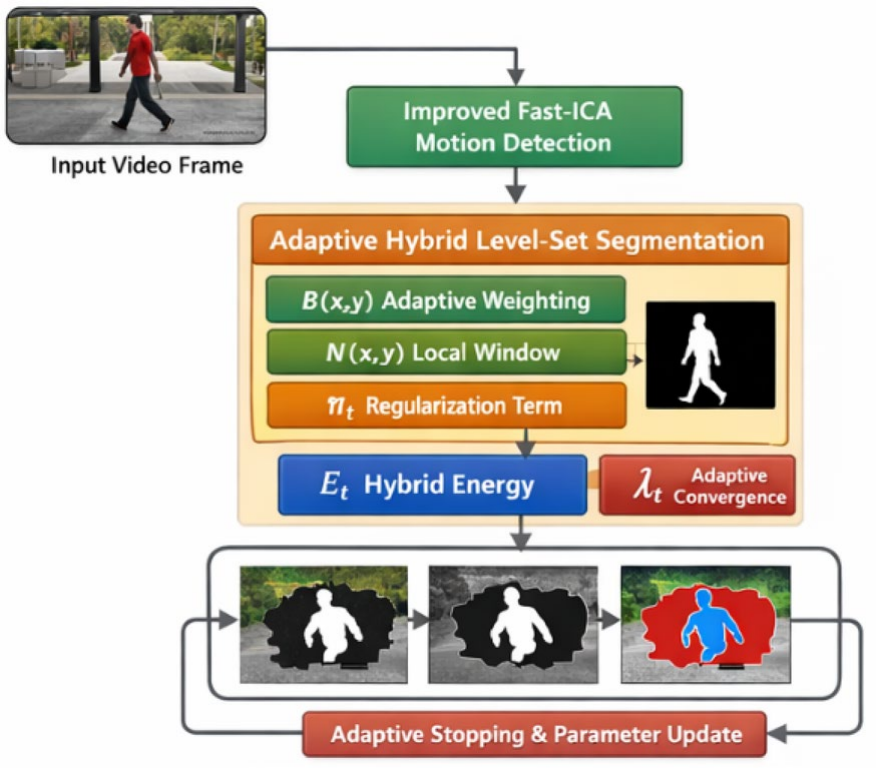
Block diagram of the proposed unsupervised motion detection via background subtraction and hybrid level set system (UMD-BHLS).

The overall system is illustrated in Fig. 1.

### Statistical motion detection

**Fig. 2:**
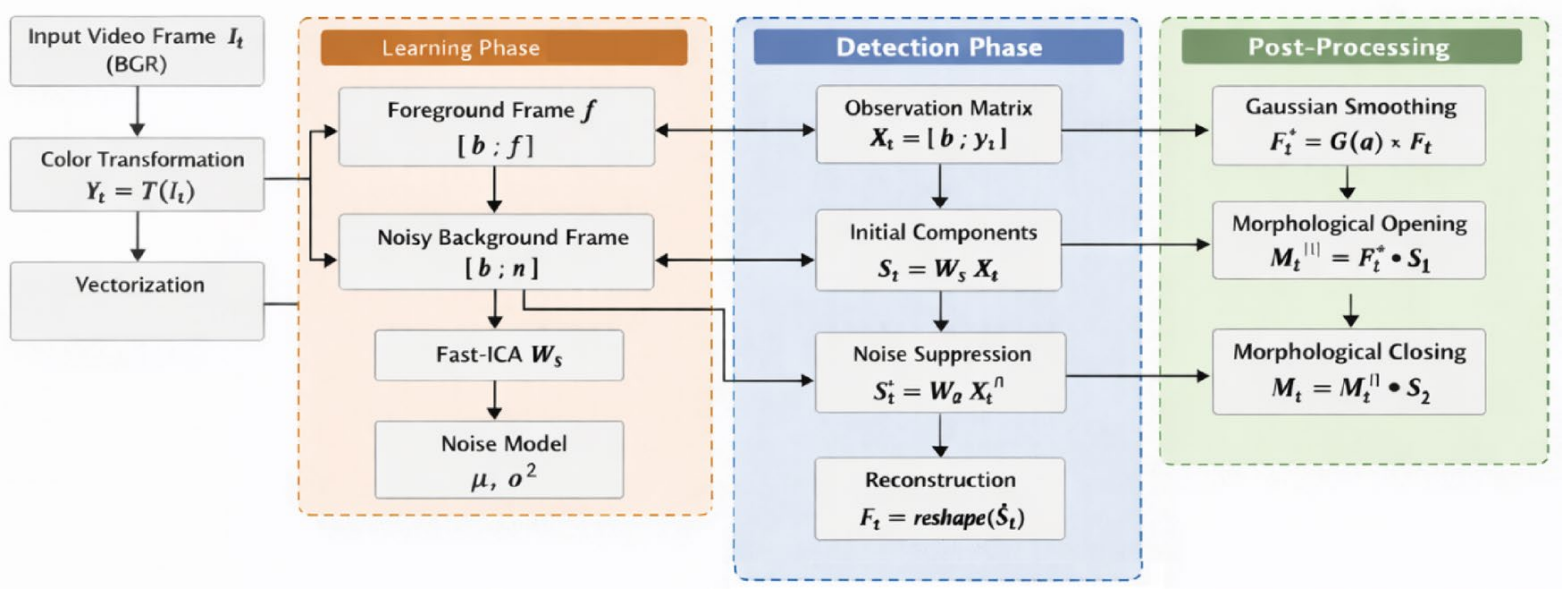
Flowchart of the enhanced Fast-ICA based motion detection phase.

In this section, the proposed method incorporates our previously developed background subtraction approach^46^ utilizing an enhanced Fast-ICA algorithm, which is used for static-camera video scenarios. Each frame is decomposed into statistically independent components. The most significant motion-related component is extracted, denoised, and converted into a binary motion mask. This mask initializes the level set function $\:{{\upphi\:}}_{0}(\mathrm{x},\mathrm{y})$ for the subsequent segmentation phase. In the following subsections, we detail the main steps of the motion detection procedure, which are outlined in Fig. 2.

#### Preprocessing

Given an input video sequence$\left\{ {{V_t}} \right\}_{{t=1}}^{T}$with frames ${I_t} \in {{\mathbb{R}}^{m \times n \times 3}}$ in the BGR color space, each frame is converted to the YC_b_C_r_ color space to separate luminance from chrominance, enhancing robustness to illumination changes and shadows. Only the luminance channel $\:{\mathrm{Y}}_{\mathrm{t}}$ is retained:

7 $${Y_t}={{\mathbb{C}}_{BGR \to Y{C_b}{C_r}}}\left( {{I_t}} \right)\left[ {:,\,:,\,1} \right]$$

where ${\mathbb{C}}\left( . \right)$ denotes the color transformation operator. The luminance channel $\:{\mathrm{Y}}_{\mathrm{t}}$ is then vectorized into a row vector of length $\:\mathrm{P}=\mathrm{m}\times\:\mathrm{n}\:$(where $\:\mathrm{m},\mathrm{n}$ are image dimensions).

8 $${y_t}=vec\left( {{Y_t}} \right) \in {{\mathbb{R}}^{1 \times P}}$$

This dimensionality reduction preserves essential structural and motion information while improving computational efficiency.

#### Learning step

Two unmixing matrices are estimated using Fast-ICA: W_s_ for foreground-background separation and W_d_ for noise suppression.

- **Foreground–background separation matrix**

Using the reference background b and a foreground-containing frame f, the observation matrix is constructed as:

9 $${X_S}=\left[ \begin{gathered} b \hfill \\ f \hfill \\ \end{gathered} \right]$$

Fast-ICA computes the unmixing matrix W_s_ by maximizing non-Gaussianity:

10 $$S={W_S}{X_S}$$

- **Noise modeling**

A second observation matrix is formed using the reference background and a noisy background frame $\:n$:

11 $${X_d}=\left[ \begin{gathered} b \hfill \\ n \hfill \\ \end{gathered} \right]$$

The noisy frame n is selected to represent typical background variations (e.g., illumination changes, moving vegetation, or camera noise). Applying Fast-ICA yields the denoising matrix W_d_.

12 $$N={W_d}{X_d}$$

The extracted noise component is modeled as a Gaussian distribution:

13 $$\mu ={\rm E}\left( N \right),\,\,\,\,\,{\sigma ^2}=Var\left( N \right)$$

#### Detection phase

For each incoming frame, the observation matrix is constructed as:

14 $${X_t}=\left[ \begin{gathered} b \hfill \\ {y_t} \hfill \\ \end{gathered} \right]$$

Applying the separation matrix gives the initial independent components:

15 $${S_t}={W_S}{X_t}$$

To suppress noise, a noise–foreground observation matrix is formed:

16 $$X_{t}^{n}=\left[ \begin{gathered} {S_t} \hfill \\ N \hfill \\ \end{gathered} \right]$$

The denoising transformation produces the refined foreground estimate:

17 $${\overset{\lower0.5em\hbox{$\smash{\scriptscriptstyle\frown}$}}{S} _t}={W_d}X_{t}^{n}$$

The denoised foreground is reshaped to image form:

18 $${F_t}=\,reshape\left( {{{\overset{\lower0.5em\hbox{$\smash{\scriptscriptstyle\frown}$}}{S} }_t}} \right)$$

#### Post-processing stage

Following the previous detection phase, a post-processing pipeline is applied to refine the resultant binary mask. This stage reduces residual noise, boundary irregularities, and minor inaccuracies in homogeneous regions. The pipeline consists of three sequential steps: adaptive Gaussian smoothing, and morphological refinement.

- **Adaptive Gaussian smoothing**

To suppress high-frequency noise while preserving structural boundaries, an adaptive Gaussian smoothing filter $\:{\mathrm{G}}_{{{\upsigma\:}}_{\mathrm{a}}}$ is applied when necessary. Unlike a fixed kernel, this filter adjusts its parameters based on the local noise $\:({\upmu\:},{{\upsigma\:}}^{2})$ estimates to prevent over-smoothing at edges. The smoothed image $F_{t}^{*}$ is obtained by:

19 $$F_{t}^{*}={F_t}*{G_{{\sigma _a}}}\left( {\mu ,\sigma } \right)$$

- **Morphological refinement**

Morphological operations are applied to correct topological imperfections such as small holes, isolated pixels, and protrusions. These operations are performed using disk-shaped structuring elements $\:{\mathrm{S}}_{1}$(for opening) and $\:{\mathrm{S}}_{2}$(for closing), with radii chosen based on image resolution and expected artifact size.

First, an opening operation removes small noise regions and thin protrusions:

20 $$M_{t}^{{\left( 1 \right)}}=F_{t}^{*} \circ \,\,{S_1}$$

Subsequently, a closing operation fills small holes and gaps within segmented regions:

21 $${M_t}=M_{t}^{{\left( 1 \right)}} \bullet \,\,{S_2}$$

where S_1_, S_2_ are structuring elements for morphological operations.

The resulting mask ${M_t}$ is further refined using a hybrid level-set evolution model for accurate object segmentation.

Unlike standard Fast-ICA, the proposed enhanced version uses dual unmixing matrices for joint foreground–background separation and noise suppression, integrates explicit Gaussian noise modeling with adaptive denoising, and includes a dedicated post-processing stage, enabling robust motion detection under dynamic backgrounds, illumination changes, and sensor noise.

### Segmentation refinement

In the refinement phase, we propose an adaptive hybrid level set energy formulation that integrates global consistency and local sensitivity through dynamically estimated parameters, ensuring robust segmentation in complex environments. Using the motion mask obtained in motion detection as the initial contour, the proposed hybrid evolution progressively refines the foreground estimate and produces the final segmentation, as detailed in Algorithm 1.

**Algorithm 1:**
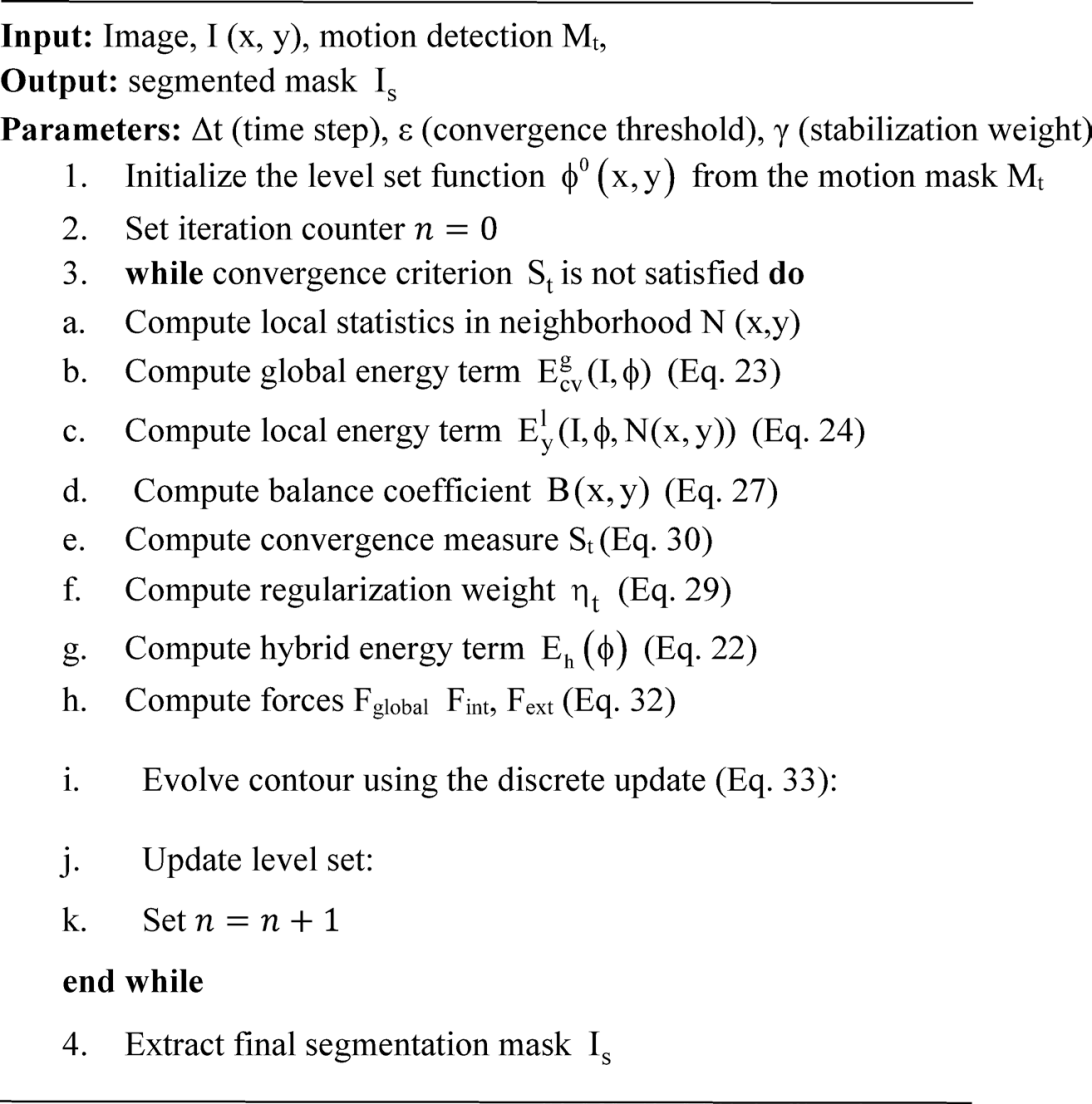
Adaptive Hybrid Level Set Evolution

#### Adaptive hybrid energy functional

The proposed fully adaptive energy integrates global consistency, local adaptivity, and temporal stability as:

22 $${E_h}\left( \phi \right)=B(x,\,y)E_{{cv}}^{g}\left( {I,\,\phi } \right)\,+\,\left( {1 - B\left( {x,y} \right)} \right).\,E_{y}^{l}\,\,\left( {I,\,\phi ,\,N\left( {x,y} \right)} \right)\,+\,{\eta _t}\,R\left( \phi \right)$$

where, $\:{\mathrm{E}}_{\mathrm{c}\mathrm{v}}^{\mathrm{g}}$ represents the global intensity fitting term, $\:{\mathrm{E}}_{\mathrm{y}}^{\mathrm{l}}$ represents the local spatially adaptive energy, $\:\mathrm{N}(\mathrm{x},\mathrm{y})$ neighborhood window, and ${\eta _t}\,R\left( \phi \right)$ is stabilization term. The scalar fields $\:\mathrm{B}(\mathrm{x},\mathrm{y})$, and $\:{{\upeta\:}}_{\mathrm{t}}$ are adaptive parameters automatically computed from image statistics to achieve local-global balance, and self-regulated stabilization, respectively. The computation details of these parameters will be shown in the next subsections.

##### Global energy term

The global component is derived from the Chan–Vese (CV) model^30^, as defined in Eq. (1). However, this formulation can lead to increased computational complexity. To mitigate this issue, we propose a simplified version inspired by the method presented in^41^. In this simplified model, the primary evolution force is given by$\,{\left| {I - \,C_{{\operatorname{int} }}^{g}} \right|^2}+\,{\left| {I - \,C_{{ext}}^{g}} \right|^2}$, which can be reformulated as $2\left( {C_{{\operatorname{int} }}^{g} - \,C_{{ext}}^{g}} \right)\left( {I - \frac{{C_{{\operatorname{int} }}^{g}+\,C_{{ext}}^{g}}}{2}} \right)$. Furthermore, the term $\frac{{C_{{\operatorname{int} }}^{g}+\,C_{{ext}}^{g}}}{2}$​​ plays a crucial role in governing pixel transitions during evolution process. By fixing$2\left( {C_{{\operatorname{int} }}^{g} - \,C_{{ext}}^{g}} \right)=1$, Eq. (1) is simplified to obtain a more efficient global fitting term:

23 $$E_{{cv}}^{g}(I,\phi )=\int\limits_{R} {\left( {I(x,y) - \frac{{C_{{_{{\operatorname{int} }}}}^{g}(x,y)+C_{{_{{ext}}}}^{g}(x,y)}}{2}} \right){H_\varepsilon }\left( \phi \right)} \,dxdy$$

**Fig. 3:**
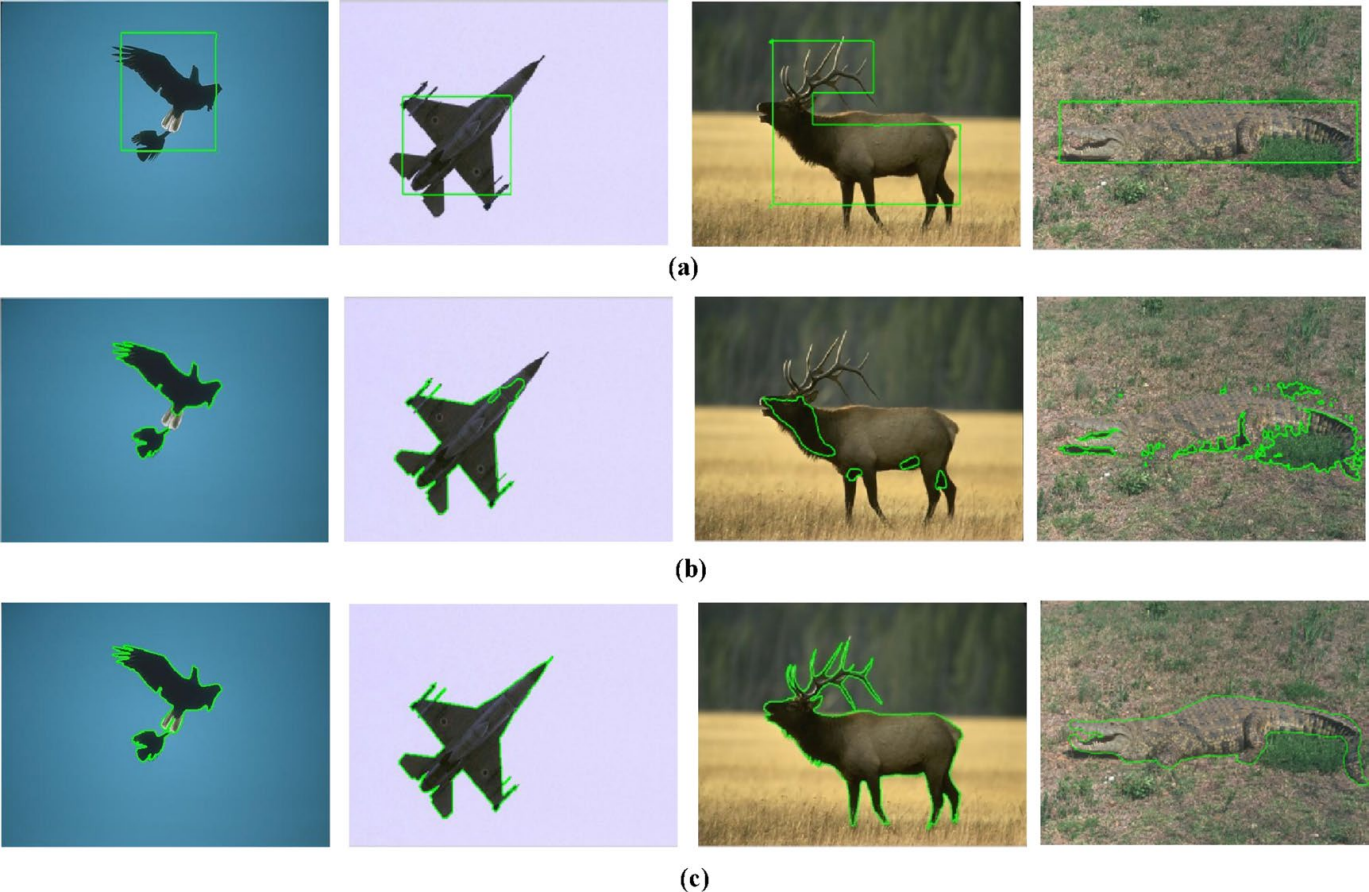
Illustration of global and local energy terms on a synthetic image^61^. (**a**) initial contour initialization, (**b**) segmentation using the global energy term, and (**c**) segmentation using the local energy term.

This global term delivers a coarse segmentation and maintains contour stability within homogeneous image backgrounds. It effectively captures the global luminance structure, thereby providing robustness against partial occlusions and moderate illumination variations. However, its discriminative power can diminish in textured or inhomogeneous regions, as illustrated in Fig. 3 b. This limitation motivates the subsequent fusion of this global model with a spatially adaptive local term to enhance segmentation accuracy across diverse image conditions.

##### Local adaptive energy

To address segmentation in spatially varying environments, a local energy term is introduced. The principal concept involves transforming the global energy functional $E_{y}^{g}$​ defined by Eq. (6) into a localized formulation, yielding a family of local energy functionals $E_{y}^{l}$​. This is accomplished by employing squared neighborhood regions N(x, y), centered at each point along the evolving contour. These localized regions facilitate the partitioning of the foreground boundary into compact interior and exterior subregions, each characterized by their respective local mean intensity values $\mu _{{int}}^{l}$ and $\mu _{{ext}}^{l}$​. The subregions size is dynamically adapted to the object’s scale and its relative position to the camera. Specifically, smaller regions are used for segmenting small, nearby objects, while larger ones are applied to capture distant or large-scale structures. This adaptive mechanism enhances the method’s ability to delineate object boundaries under varying scale and perspective conditions, the local component effectively handles non-uniform illumination, local contrast variations, and fine object details that are typically overlooked by global methods, as shown in Fig. 3 c. It is formulated as follows:

24 $$E_{y}^{l}(\mu _{{\operatorname{int} }}^{l},\,\mu _{{ext}}^{l},\phi )= - \frac{1}{2}{\left( {\mu _{{\operatorname{int} }}^{l} - \,\mu _{{ext}}^{l}} \right)^2}$$

The localized formulation can be interpreted as a spatially constrained version of the global Yezzi energy, obtained by replacing the global means $C_{{int}}^{g}$ and $C_{{ext}}^{g}$with locally estimated statistics $\mu _{{int}}^{l}$ and $\mu _{{ext}}^{l}$computed within a neighborhood window $\:N\left(x\right)$ as:

25 $$\mu _{{\operatorname{int} }}^{l}(\phi )=\frac{{\int\limits_{{{N_{\operatorname{int} }}}} {{\mathbf{I}}\,(x,y){H_\varepsilon }\left( \phi \right)N(x,y)\,dxdy} }}{{\int\limits_{{{N_{\operatorname{int} }}}} {{H_\varepsilon }\left( \phi \right)N(x,y)\,dxdy} }}$$

26 $$\mu _{{ext}}^{l}(\phi )=\frac{{\int\limits_{{{N_{ext}}}} {{\mathbf{I}}\,(x,y)\left( {1 - {H_\varepsilon }\left( \phi \right)} \right)N(x,y)\,dxdy} }}{{\int\limits_{{{N_{ext}}}} {\left( {1 - {H_\varepsilon }\left( \phi \right)} \right)N(x,y)\,dxdy} }}$$

To avoid unnecessary computations across the entire domain Ω, we implement a narrow band strategy, restricting the evolution of the level set function to a thin region TR surrounding the zero-level set. This dramatically improves efficiency while preserving segmentation accuracy.

##### Adaptive global–local balancing coefficient

To enable automatic regulation between global and local energy terms, we introduce an entropy-driven adaptive balance coefficient defined as:

27 $$B\,(x,y)=\frac{1}{{1+\exp \left( { - \gamma \left( {{E_n}\left( {x,y} \right) - {\mu _E}} \right)} \right)}}$$

where $\:{\mathrm{E}}_{\mathrm{n}}(\mathrm{x},\mathrm{y})$ is the normalized local entropy or gradient energy, $\:{{\upmu\:}}_{\mathrm{E}}$ is its mean, and the parameter $\:{\upgamma\:}$ controls the sensitivity of the weighting. This formulation ensures a context-dependent balance: in uniform or homogeneous regions where $\:{\mathrm{E}}_{\mathrm{n}}(\mathrm{x},\mathrm{y})\approx\:{{\upmu\:}}_{\mathrm{E}}$, the coefficient $\:\mathrm{B}$ approaches 1. This prioritizes the global energy term, promoting smooth and stable evolution. Conversely, in textured or high-frequency regions, $\:\mathrm{B}$ tends toward 0, allowing the more discriminative local energy term to guide the contour with greater precision. This self-adaptive weighting eliminates the need for manual parameter tuning and significantly enhances the model’s adaptability to scenes of varying complexity.

##### Adaptive stabilization term

To ensure geometric regularity while preserving the contour’s capacity for necessary topological adaptation, a curvature-based stabilization or regularization term is incorporated into the energy functional. This term penalizes excessive length and high curvature, promoting smooth segmentation boundaries.

28 $$R(\phi )=\int\limits_{\Omega } {{{\left| {\nabla \phi } \right|}^2}\,dxdy}$$

The influence of regularization is dynamically controlled via a temporally adaptive weight ${\eta _t}$, defined as:

29 $${\eta _{t+1}}=\left\{ {\begin{array}{*{20}{c}} {\,{\eta _t} - \,{\eta _t}*(1 - {S_t})\,\,\,if\,\,{S_t}>\,\,{S_{t - 1}}} \\ {\,{\eta _t}+\,{\eta _t}*(1 - {S_t})\,\,\,if\,\,{S_t}<\,\,\,{S_{t - 1}}} \end{array}} \right.$$

where $\:{\mathrm{S}}_{\mathrm{t}}$ is the expression of the self-regulating convergence criterion or stopping criterion:

30 $$\mathop s\nolimits_{t} =\frac{{2\int {{\phi _t}(x,\,y).{\phi _{t - 1}}(x,\,y)dxdy} }}{{\int {{\phi _t}(x,\,y)dxdy} +\int {{\phi _{t - 1}}(x,\,y)dxdy} }} \geqslant \lambda$$

**Fig. 4:**
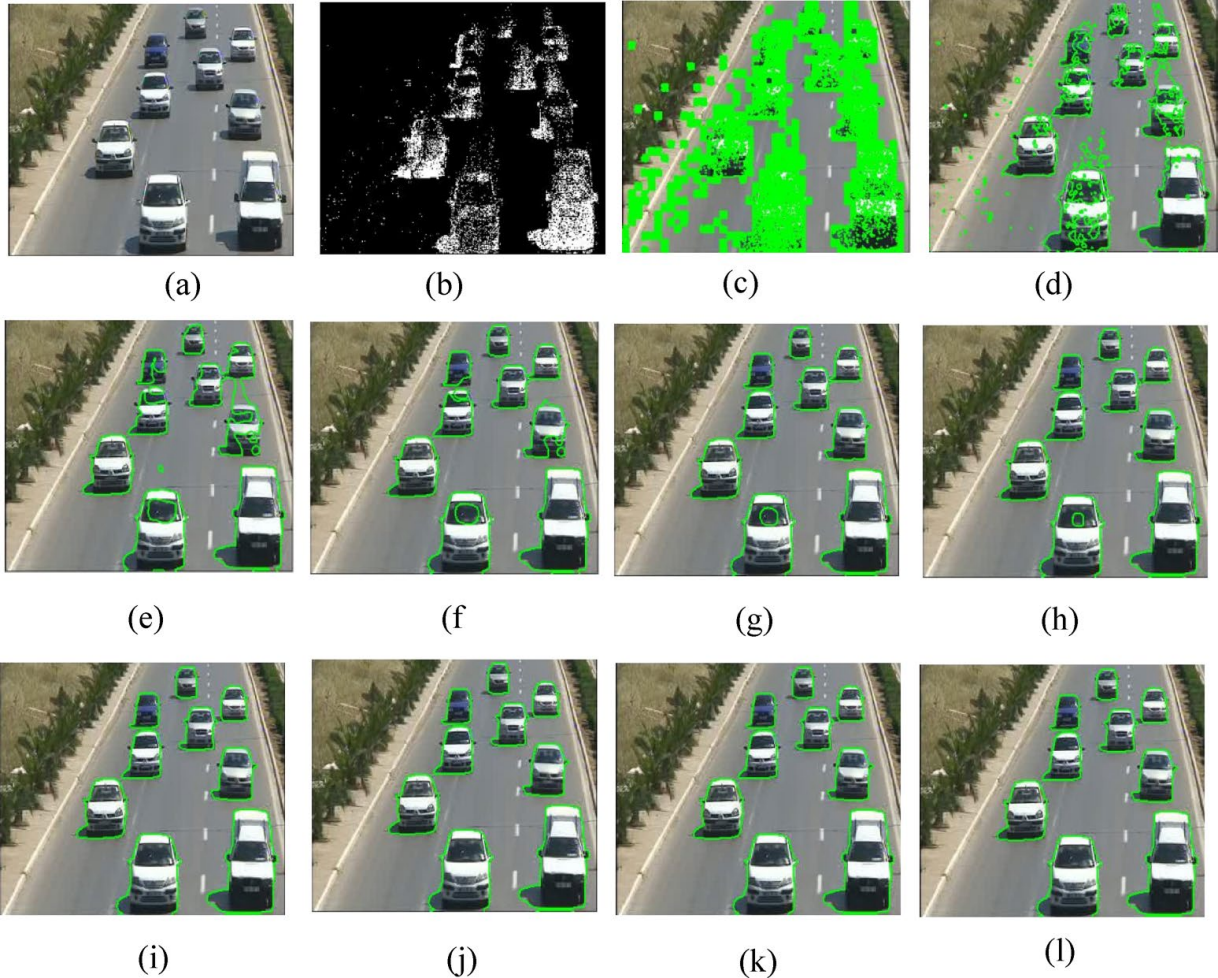
Temporal evolution of the segmentation process driven by the adaptive stabilization term. (**a**) Original frame. (**b**) Initial mask obtained from Fast-ICA motion detection. (**c**–**l**) Contour evolution at iterations: (**c**) *n* = 1, (**d**) *n* = 20, (**e**) *n* = 100, (**f**) *n* = 200, (**g**) *n* = 300, (**h**) *n* = 400, (**i**) *n* = 535, (**j**) *n* = 1000, (**k**) *n* = 1500, (**l**) *n* = 1800.

Where $\lambda$ is the convergence threshold, the regularization weight ${\eta _t}$is adaptively controlled by the convergence measure ${S_t}$. When ${S_t}$ increases, indicating that the evolving contour is approaching object boundaries, the regularization effect is attenuated to avoid excessive smoothing and to improve boundary localization, as illustrated in the later stages of (Fig. 4 g-l). In contrast, when ${S_t}$decreases during the early evolution or when the contour remains far from the target boundaries (Fig. 4 c-f), stronger regularization is enforced to encourage smooth propagation, suppress noise, and accelerate convergence. This self-regulating mechanism is closely related to curvature-driven flows, in which diffusion strength is reduced near stable interfaces and increased in homogeneous regions, thereby ensuring numerical stability while preserving accurate contour delineation.

##### Adaptive evolution equation

Using variational principles, the proposed adaptive hybrid energy functional in (22) is minimized through gradient descent on the level set function $\phi$. This yields a Hamilton–Jacobi partial differential equation that governs the dynamic evolution of the contour:

31 $$\frac{{\partial \phi }}{{\partial t}}=\,{F_h}(\phi )= - {\delta _\varepsilon }\left( \phi \right)\left[ {B.\,F_{{global}}^{{}} - \left( {1 - B} \right)\left( {\left( {\mu _{{\operatorname{int} }}^{l}(\phi ) - \,\mu _{{ext}}^{l}(\phi )} \right).\left( {F_{{\operatorname{int} }}^{l}(\phi ) - \,F_{{ext}}^{l}(\phi )} \right)} \right)} \right]+{\eta _t}(\phi )\nabla \phi$$

The terms $F_{{global}}^{{}}\,$ correspond to the global Chan–Vese-driven force, $F_{{\operatorname{int} }}^{l}\,$ and $\,F_{{ext}}^{l}$ represent the locally adaptive interior and exterior Yezzi-driven forces, respectively, as defined in (32). The stabilization term ${\eta _t}\nabla \phi$enforces smoothness of the evolving contour while preserving topological flexibility.

32 $$\left\{ \begin{gathered} F_{{global}}^{{}}=\left( {I(x,y) - \frac{{C_{{\operatorname{int} }}^{g}+\,C_{{ext}}^{g}}}{2}} \right) \hfill \\ F_{{\operatorname{int} }}^{l}=\frac{{I(x,y) - \mu _{{\operatorname{int} }}^{l}}}{{\int\limits_{{{N_{\operatorname{int} }}}} {{H_\varepsilon }\left( \phi \right)N(x,y)\,dxdy} }} \hfill \\ F_{{ext}}^{l}=\frac{{I(x,y) - \mu _{{ext}}^{l}}}{{\int\limits_{{{N_{ext}}}} {\left( {1 - {H_\varepsilon }\left( \phi \right)} \right)N(x,y)\,dxdy} }} \hfill \\ \end{gathered} \right.$$

For numerical implementation, the continuous evolution equation in (31) is discretized in time using an explicit Euler scheme, yielding the following iterative update of the level set function:

33 $$\phi _{{i,j}}^{{n+1}}=\phi _{{i,j}}^{n}+\Delta t\,F_{h}^{{}}\left( {\phi _{{i,j}}^{n}} \right)$$

where $F_{h}^{{}}\left( {\phi _{{i,j}}^{n}} \right)$ is defined as:

34 $$F_{h}^{{}}\left( {\phi _{{i,j}}^{n}} \right)= - {\delta _\varepsilon }\left( {\phi {}_{{i,j}}} \right)\left[ {B.\,F_{{global}}^{{}}\left( {\phi _{{i,j}}^{n}} \right) - \left( {1 - B} \right)\left( {\left( {\mu _{{\operatorname{int} }}^{l}\left( {\phi _{{i,j}}^{n}} \right) - \,\mu _{{ext}}^{l}\left( {\phi _{{i,j}}^{n}} \right)} \right).\left( {F_{{\operatorname{int} }}^{l}\left( {\phi _{{i,j}}^{n}} \right) - \,F_{{ext}}^{l}\left( {\phi _{{i,j}}^{n}} \right)} \right)} \right)} \right]+{\eta _n}\nabla \phi _{{i,j}}^{n}$$

and$\Delta t$is the artificial time step controlling the numerical stability and convergence speed of the level set evolution. In all experiments, $\Delta t$is chosen to satisfy the Courant–Friedrichs–Lewy (CFL)^32^ condition to ensure stable evolution. ${\delta _\varepsilon }\left( . \right)$denotes the regularized Dirac delta function that confines the evolution to a narrow band around the zero-level set, improving numerical efficiency. The coefficient $B\left( {x,y} \right)$adaptively balances the influence of the global and local forces according to local image statistics.

This adaptive evolution formulation enables stable convergence in homogeneous regions while preserving fine boundary details in textured or low-contrast areas. The proposed hybrid evolution mechanism also improves robustness when the initial motion mask obtained from Fast-ICA is imperfect. In such situations, the local Yezzi energy term guides contour evolution using localized image statistics, allowing the contour to expand toward foreground regions even when the initial mask is partially incomplete. Meanwhile, the global Chan–Vese component maintains regional consistency and suppresses noise artifacts. Through the proposed adaptive energy balancing mechanism, the relative contribution of these components is dynamically adjusted during contour evolution, enabling stable and accurate foreground segmentation even under imperfect initialization conditions.

## Experimental results

This section presents a comprehensive experimental evaluation of the proposed UMD-BHLS framework, assessing its effectiveness, robustness, and computational efficiency in comparison with representative several state-of-the-art methods. All experiments were conducted under consistent conditions to ensure a fair and reproducible assessment.

### Dataset description

The proposed framework was evaluated on the CDnet-2014 benchmark dataset^62^, which is widely recognized as one of the most challenging and comprehensive benchmarks for motion detection and change analysis. CDnet-2014 extends the earlier CDnet-2012 dataset and comprises 31 video sequences totaling more than 70,000 frames, with individual sequences ranging from 900 to over 7,000 frames. The dataset is organized into 11 challenging categories, designed to reflect real-world surveillance conditions: Baseline (BSL): static background scenes, Dynamic Background (DBA): moving foliage, water surfaces, Camera Jitter (CJI): global camera motion, Shadow (SHW): illumination gradients and cast shadows, Intermittent Object Motion (IOM): temporarily static foreground objects, Thermal (THL): infrared imagery, Bad Weather (BWT): rain and snow, Low Frame Rate (LFR): motion aliasing effects, Night Videos (NV): poor illumination and artificial lighting, PTZ: pan–tilt–zoom camera motion, Turbulence (TRL): atmospheric heat distortion.

The PTZ category involves explicit camera motion compensation, which falls outside the scope of static-camera background subtraction. Following common practice, it is excluded from the evaluation.

### Evaluation metrics

Performance is quantitatively assessed using the standard Recall (Re), Precision (Pr), and F-measure metrics, computed at the pixel level:

- Recall measures the proportion of correctly detected foreground pixels,
- Precision evaluates the robustness against false positives, and.
- F-measure provides a balanced indicator of segmentation quality through their harmonic mean.

These metrics are defined as^62^:

35 $$\begin{gathered} \operatorname{Re} call=\frac{{TP}}{{TP+FN}},\,\Pr ecision=\frac{{TP}}{{TP+FP}} \hfill \\ \hfill \\ \end{gathered}$$

36 $$F - measure=2\frac{{\operatorname{Re} call \times \Pr ecision}}{{\operatorname{Re} call+\Pr ecision}}$$

where TP, FP, and FN denote the numbers of True Positives, False Positives, and False Negatives pixels, respectively.

Segmentation performance often requires a delicate balance between recall and precision. High recall values are often obtained by detecting a greater number of foreground pixels; however, this can inadvertently lead to an increase in false positives, thereby decreasing precision. An effective segmentation algorithm must, therefore, maintain a high recall without significantly compromising precision. As a harmonized metric of recall and precision, the F-measure effectively serves as a balanced indicator of overall segmentation quality. In accordance with the standard CDNet-2014 evaluation protocol^62^, all metrics are computed at the pixel level for each frame, then averaged across all frames within a given video sequence, and finally averaged across sequences belonging to the same category. This hierarchical averaging yields statistically robust performance indicators and mitigates the influence of sequence-specific anomalies or outliers.

### Parameter settings

The proposed UMD-BHLS framework is designed to minimize dependence on manual parameter tuning through fully adaptive mechanisms embedded in the energy formulation and evolution process. In particular, the global–local balancing coefficient $B\,(x,y)$, and the regularization weight ${\eta _t}$ are automatically estimated from intrinsic image statistics, including local entropy, gradient magnitude, and convergence behavior, as detailed in Sect. 3. In addition to these adaptive components, a limited number of numerical parameters are required for implementation purposes, such as time discretization and smoothing constants. These parameters are fixed across all experiments and follow standard values commonly adopted in Chan–Vese and Yezzi-based level set models. All experiments were conducted using the original resolutions provided by the CDNet-2014 dataset, without additional resizing or cropping, to ensure consistent evaluation conditions across sequences. For supervised and semi-supervised baseline methods, we report the results provided by the official CDNet-2014 benchmark and the corresponding original publications, and no retraining or additional parameter tuning was performed in this work. Table 1 summarizes the default parameter values used throughout this work.

**Table 1:** Default numerical parameters of the proposed UMD-BHLS method.

Parameter	Description	Value
S_1_	Structuring element (opening) (Eq. 20)	Disk, radius 2
S_2_	Structuring element (closing) (Eq. 21)	Disk, radius 3
$\Delta t$	Time step of level set evolution (explicit Euler)	0.1
_N(x, y)_	Local window size	5*5
$\gamma$	Sensitivity factor in (Eq. 27)	5
$\lambda$	Convergence threshold (Eq. 30)	10^− 4^

To further analyze the contribution of the proposed hybrid formulation, the following subsection compares UMD-BHLS method directly with its foundational Global Chan–Vese and Local Yezzi component models.

### Quantitative analysis

#### Comparison with foundational component models

This subsection presents a quantitative comparison between the proposed UMD-BHLS framework and its two foundational region-based active contour models: the Global Chan–Vese (CV) model and the Local Yezzi-based model. The objective is to assess the individual limitations of purely global and purely local formulations and to evaluate the extent to which their adaptive integration improves motion segmentation performance.

**Table 2:** Quantitative comparison of the proposed UMD-BHLS ($E_{h}^{{}}$) method with Global CV ($E_{{CV}}^{g}$) and Local Yezzi ($E_{y}^{l}$) models on the CDNet-2014 benchmark.

Method Category	Global CV:$E_{{CV}}^{g}$			Local Yezzi:$E_{y}^{l}$			UMD-BHLS:$E_{h}^{{}}$		
	Re	Pr	F-measure	Re	Pr	F-measure	Re	Pr	F-measure
BSL	0.9522	0.5948	0.7278	0.9650	0.8957	0.9244	**0.9811**	**0.9193**	**0.9489**
DBA	0.9202	0.5732	0.7018	0.9509	0.8049	0.8609	**0.9846**	**0.8877**	**0.9341**
CJI	0.9667	0.7063	0.8093	0.9606	0.8268	0.8828	**0.9693**	**0.9038**	**0.9348**
SHW	0.9164	0.7375	0.8160	0.9443	0.8923	0.9146	**0.9570**	**0.9309**	**0.9370**
IOM	0.9622	0.7044	0.8044	0.9749	0.8815	0.9233	**0.9804**	**0.9050**	**0.9409**
THL	0.9328	0.7778	0.8433	0.9328	0.9044	0.9171	**0.9398**	**0.9087**	**0.9224**
BWT	0.9414	0.8146	0.8717	0.975	0.9050	0.9315	**0.9901**	**0.9407**	**0.9622**
LFR	0.9633	0.6509	0.7742	0.9738	0.8340	0.8948	**0.9820**	**0.8868**	**0.9251**
NV	0.9311	0.6250	0.7413	0.9533	0.8020	0.8606	**0.9445**	**0.8435**	**0.8833**
TRL	**0.9608**	0.7041	0.8044	0.8642	0.9339	0.8907	0.8851	**0.9627**	**0.9219**
Overall	0.9447	0.6888	0.7894	0.9494	0.8680	0.9000	0.9613	0.9089	0.9310

**Fig. 5:**
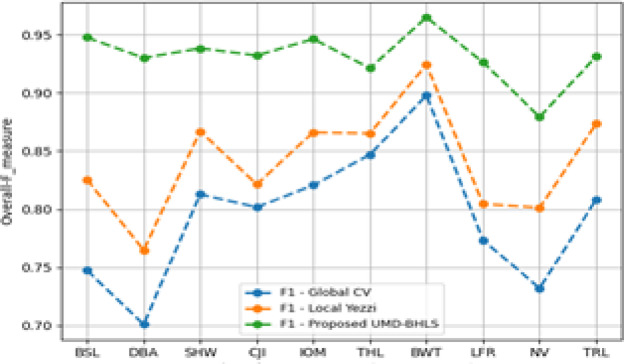
Per-category F-measure on the CDnet-2014 dataset for the three compared models: global Chan-Vese (blue), local region-based model (orange), and the proposed UMD-BHLS (green).

Table 2 encapsulates the quantitative results for ten categories from the CDnet2014 dataset for the three segmentation models: Global CV (Chan-Vese), Local Yezzi, and the proposed UMD-BHLS model. The reported performance metrics recall, precision, and F-measure are expressed as mean values computed across all frames within the test sequences, thus offering a statistically robust evaluation of each model’s effectiveness. Quantitative results show that our model consistently outperforms both Global CV and Local Yezzi. It attains an average recall of 0.9613, precision of 0.9089, and an F-measure of 0.9310, outperforming both individual global and local approaches. The global CV model, while achieving a high recall of 0.9447, suffers from a lower precision of 0.6888, yielding an F-measure of 0.7894. In contrast, the local Yezzi model presents a more favorable balance, reaching 0.9494 recall, 0.8680 precision, and an F-measure of 0.900. The F-measure reveals the advantage of the proposed hybrid segmentation model that integrates the strengths of both global and local active contour formulations, as shown in Fig. 5. It achieves high F-measure values across most categories and peaking in challenging scenes like Bad Weather. In contrast, the local Yezzi model outperforms the global CV method due to its use of local region information, but it has limitations in more challenging conditions such as Night Videos, Dynamic Background, and Low Frame Rate categories. Overall, the global CV model presents the weakest performance and highlights its limited adaptability to local appearance changes and motion features.

Category-wise analysis further confirms the robustness of the proposed approach. In BSL and DBA categories, UMD-BHLS reduces background clutter and repetitive motion artifacts, yielding substantial precision gains compared to the Global CV model. In shadow and camera jitter scenarios, the UMD-BHLS formulation maintains stable contours and avoids shadow leakage, achieving F-measure values consistently above 0.93. Moreover, in challenging conditions such as bad weather, low frame rate, night videos, and turbulence, UMD-BHLS demonstrates strong resilience, whereas both foundational models exhibit notable performance degradation. These results visually confirm the quantitative analysis, highlighting the stability and robustness achieved through the adaptive global–local energy integration.

#### Comparative analysis with state-of-the-art methods

**Fig. 6:**
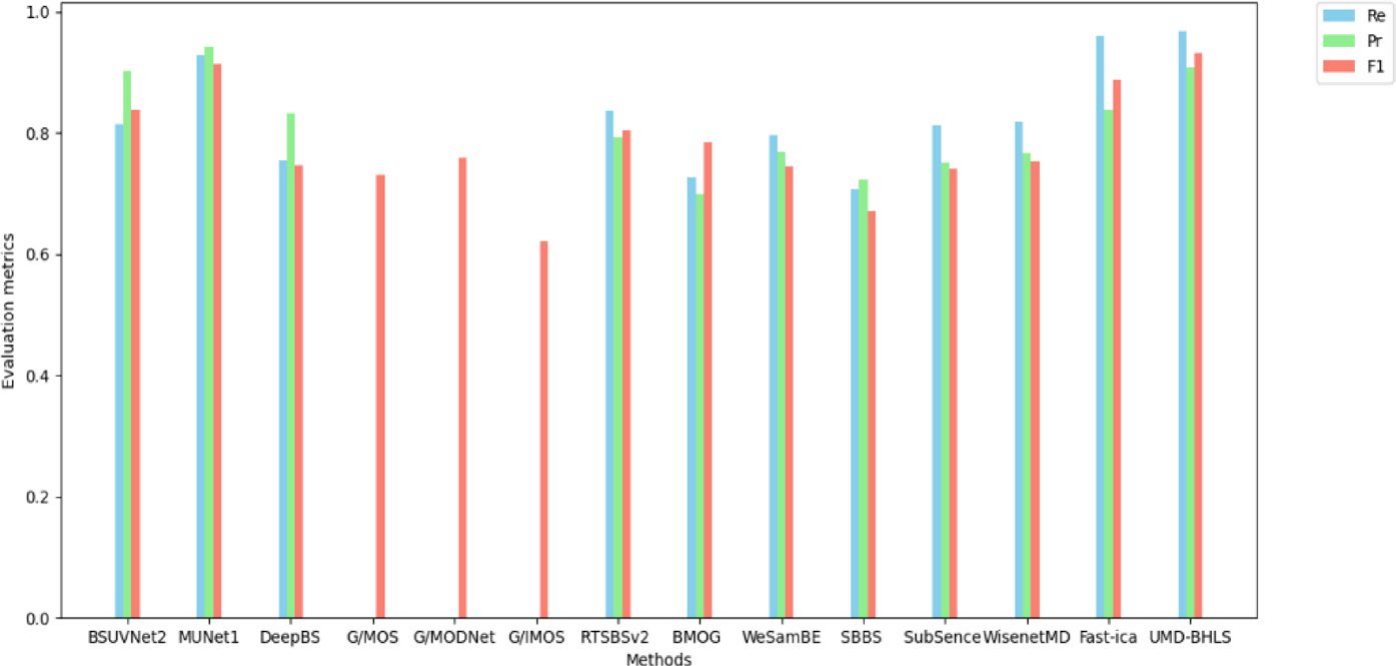
Overall F-measure of the proposed UMD-BHLS compared to state-of-the-art unsupervised, semi-supervised and supervised methods on CDnet-2014. Average values computed across all frames in each category.

This subsection presents a rigorous comparative evaluation of the proposed UMD-BHLS framework against a broad range of representative state-of-the-art motion detection approaches on the challenging CDnet-2014 benchmark dataset. Table 3 summarizes the overall performance in terms of Recall (Re), Precision (Pr), and F-measure across the benchmark. The UMD-BHLS achieves the highest overall F-measure, outperforming all compared methods, as illustrated in the bar chart comparison of Fig. 6. This result is underpinned by an exceptional balance between high recall and high precision), indicating both excellent foreground detection capability and effective suppression of false positives.

**Table 3:** Average Recall, Precision, and F-measure comparisons of multiple BGS algorithms from CDnet-2014.

Method Metrics	Supervised			Semi-supervised			Unsupervised							
	BSUV -Net^14^	MU-Net1 ^15^	DeepBS ^16^	Graph- MOS^57^	GraphMOD-Net^55^	Graph-IMOS^56^	RT-SBS^25^	BMOG ^19^	WeSamBE ^23^	SBBS ^20^	SuBSENSE ^22^	WisenetMD ^24^	Enhanced Fast-ICA^46^	UMD-BHLS (our)
Re	0.8136	0.9277	0.7545	-	-	-	0.8361	0.7265	0.7955	0.7073	0.8124	0.8179	0.9605	**0.9613**
Pr	0.9011	0.9414	0.8332	-	-	-	0.7743	0.6981	0.7679	0.7221	0.7509	0.7668	0.8373	**0.9089**
F-Measure	0.8844	0.9147	0.7458	0.7302	0.7593	0.6211	0.8279	0.7836	0.8608	0.7403	0.8619	0.7535	0.8887	**0.9310**

To provide deeper insight into these results, we next discuss the performance of competing approaches according to their methodological category.

#### Unsupervised traditional methods

 Classical background subtraction techniques such as RT-SBS^25^, BMOG^19^, WeSamBE^23^, SBBS^20^, SuBSENSE^22^, and WisenetMD^24^ demonstrate competitive performance in controlled environments but exhibit notable limitations under complex conditions. These methods often struggle with dynamic backgrounds, illumination changes, and noise, leading to increased false positives or fragmented segmentations. In contrast, UMD-BHLS consistently surpasses these approaches across all benchmark categories, demonstrating that the integration of statistical source separation with adaptive geometric refinement yields substantially improved segmentation coherence and boundary precision. The proposed framework effectively mitigates common failure modes of classical BGS algorithms, such as over-segmentation, ghost artifacts, and fragmented foreground masks.

#### Semi-supervised graph-based methods

 Recent graph-based segmentation methods, including GraphMOS^57^, GraphMOD-Net^55^, and GraphIMOS^56^, exploit spatial–temporal graph structures to enhance motion segmentation while reducing dependence on fully annotated training data. Although these models provide promising results in certain scenarios, their overall performance remains significantly below that of UMD-BHLS, particularly in challenging categories involving severe background instability, turbulence, and low-frame-rate motion aliasing.

#### Supervised deep learning models

 Supervised methods such as BSUV-Net 2.0^14^, MU-Net1^15^, and DeepBS^16^ achieve strong performance by learning semantic foreground features. However, they require large annotated datasets and high computational resources. In contrast, UMD-BHLS attains competitive accuracy while remaining fully unsupervised and more suitable for practical surveillance deployment. These comparisons highlight that the superior performance of UMD-BHLS stems from its adaptive contour-driven refinement mechanism, which preserves spatial continuity and maintains robustness across diverse surveillance conditions.

To provide deeper insight into performance consistency, Table 4 reports the detailed F-measure scores across individual CDnet-2014 categories. UMD-BHLS demonstrates uniformly strong robustness across nearly all challenging scenarios, achieving particularly high accuracy in DBA (0.9341), BWT (0.9622), LFR (0.9251), and NV (0.8833), confirming its resilience to illumination degradation, atmospheric noise, repetitive motion patterns, and temporal sampling limitations. Although the enhanced Fast-ICA method attains a slightly higher score in the CJ category, UMD-BHLS maintains competitive performance without significant degradation, indicating strong stability under moderate global camera motion disturbances. This demonstrates that the integration of statistical Fast-ICA with adaptive hybrid level sets delivers state-of-the-art accuracy while maintaining practical advantages: unsupervised operation, real-time capability, and strong generalization without dataset dependency.

**Table 4:** F-measure comparison of the proposed UMD-BHLS method against multiple state-of-the-art BGS algorithms on the CDnet- 2014 benchmark.

Method		BSL	DBA	SHW	CJI	IOM	THL	BWT	LFR	NV	TRL	Overall
Unsupervised	RT-SBS^25^	0.9535	0.9497	0.9217	0.8233	0.8946	0.8697	0.8279	0.7341	0.5629	0.7315	0.8045
	BMOG^19^	0.8301	0.8414	0.7928	0.7493	0.5291	0.6348	0.7836	0.6102	0.4982	0.6932	0.7836
	WeSamBE^23^	0.9413	0.8686	0.7440	0.7976	0.7392	0.7962	0.8608	0.6602	0.5929	0.7737	0.7446
	SBBS^20^	0.9192	0.7105	0.8128	0.7347	0.6795	0.7499	0.7403	0.5534	0.5055	0.7362	0.6711
	SuBSENSE^22^	0.9503	0.8646	0.8128	0.8152	0.6569	0.8171	0.8619	0.6445	0.5599	0.7792	0.7408
	WisenetMD^24^	0.9487	0.8376	0.8984	0.8228	0.7264	0.8152	0.8616	0.6404	0.5701	0.8304	0.7535
	Enhanced Fast-ICA^46^	0.8963	0.9507	0.8890	0.9649	0.8756	0.9087	**0.9701**	0.8717	0.8532	0.8701	0.8887
	**UMD-BHLS (our)**	0.9489	0.9341	0.9370	0.9348	0.9409	0.9224	0.9622	**0.9251**	**0.8833**	**0.9219**	**0.9310**
Semi-supervised	GraphMOS^57^	0.9398	0.9653	0.7334	0.7005	0.3607	0.7292	0.8294	0.5538	**0.8211**	**0.8233**	0.7302
	GraphMOD-Net^55^	0.9550	0.9420	0.8510	0.7200	0.5540	0.6820	0.8390	0.5210	-	-	0.7593
	GraphIMOS^56^	0.7003	0.6807	0.5868	0.6700	0.5284	0.6453	0.6377	0.5478	-	-	0.6211
Supervised	BSUV-Net 2.0^55^	0.9620	0.9562	0.9057	0.9004	0.8263	0.8932	0.8844	0.7902	0.5857	0.8174	0.8387
	MU-Net1 ^56^	**0.9875**	**0.9729**	**0.9836**	**0.9802**	**0.9872**	**0.9825**	0.9319	0.7237	0.8575	0.8499	0.9147
	DeepBS^57^	0.9580	0.9092	0.8761	0.8990	0.6098	0.7583	0.8301	0.6002	0.5835	0.8455	0.7458

### Computational performance and real-time capability

The computational efficiency of the proposed UMD-BHLS framework is evaluated to assess its suitability for real-time intelligent video surveillance applications. Experiments were conducted on a standard desktop platform with an Intel Core i7 CPU, 8 GB RAM, running MATLAB without GPU acceleration. The method processes 320 × 240 sequences at an average rate of 29 Frame-Per-Second (FPS) and 720 × 480 sequences at approximately 20 FPS, satisfying practical real-time constraints for surveillance applications. Compared with widely used background subtraction baselines, the proposed framework achieves competitive runtime while operating entirely on CPU hardware, whereas many recent deep learning–based approaches typically require GPU acceleration to achieve better processing speeds. Runtime analysis indicates that the Fast-ICA–based motion detection stage dominates the computational cost, accounting for approximately 70–80% of the total runtime. This stage performs the core statistical source separation and the initial foreground extraction. The remaining 20–30% corresponds to the hybrid level-set refinement stage, which iteratively refines object boundaries, suppresses residual artifacts, and enhances segmentation precision. The level-set refinement remains computationally efficient due to two design mechanisms: (i) a narrow-band formulation, which restricts computations to pixels near detected foreground boundaries, and (ii) an adaptive convergence criterion (Eq. 30) that terminates contour evolution once updates become negligible. Across the CDNet-2014 categories, convergence typically occurs within a moderate number of iterations with limited variation depending on scene complexity.

In practice, the computational cost exhibits an approximately linear trend with respect to image resolution and sequence length in the tested configurations, as the initialization is performed once per frame while refinement is restricted to localized contour regions. This observation is empirical and does not constitute a formal complexity analysis.

### Ablation study

An ablation study was conducted on the CDnet-2014 benchmark to quantitatively assess the contribution of each component within the proposed UMD-BHLS framework and to highlight the effectiveness of integrating statistical motion separation with adaptive contour refinement. Several reduced configurations were evaluated under identical experimental conditions using the standard Recall (Re), Precision (Pr), and F-measure metrics averaged across all dataset categories, including: (i) the Fast-ICA-only baseline, which performs motion detection solely through statistical independence analysis; (ii) Fast-ICA combined with a purely global Chan–Vese refinement term; (iii) Fast-ICA combined with a purely local Yezzi-based refinement term; and (iv) the complete UMD-BHLS model integrating both global and local energies through adaptive balancing and self-regulated regularization.

**Table 5:** Ablation results on CDnet-2014.

Configuration	Re	Pr	F-measure
Fast-ICA-Only	0.9605	0.8373	0.8887
Fast-ICA + Global CV	0.9447	0.6888	0.7894
Fast-ICA + Local Yezzi	0.9494	0.8680	0.9000
UMD-BHLS (full)	**0.9613**	**0.9089**	**0.9310**

The quantitative results reported in Table 5 clearly demonstrate that each component plays a critical role in achieving robust segmentation performance. The Fast-ICA-only configuration achieves a high recall of 0.9605, confirming its strong sensitivity in detecting foreground motion, yet its lower precision of 0.8373 reflects susceptibility to noise and dynamic background interference due to the absence of spatial refinement. Incorporating only the global Chan-Vese term significantly reduces performance, yielding the lowest F-measure of 0.7894, as global homogeneity assumptions are frequently violated in complex surveillance scenes characterized by shadows, textured backgrounds, and illumination variations. The local Yezzi-based refinement improves robustness compared to the global-only variant, achieving an F-measure of 0.9000, but remains limited in highly noisy environments where unstable local statistics can lead to fragmented contours. In contrast, the full UMD-BHLS framework achieves the best overall performance with Re = 0.9613, Pr = 0.9089, and F- measure = 0.9310, demonstrating that the adaptive integration of global consistency and local boundary sensitivity is essential for balanced and accurate motion segmentation. Overall, the ablation results confirm that the proposed hybrid statistical–geometric design, coupled with self-adaptive parameter regulation, provides substantial gains over simplified variants and is necessary to achieve state-of-the-art robustness across diverse surveillance conditions.

### Qualitative analysis

**Fig. 7:**
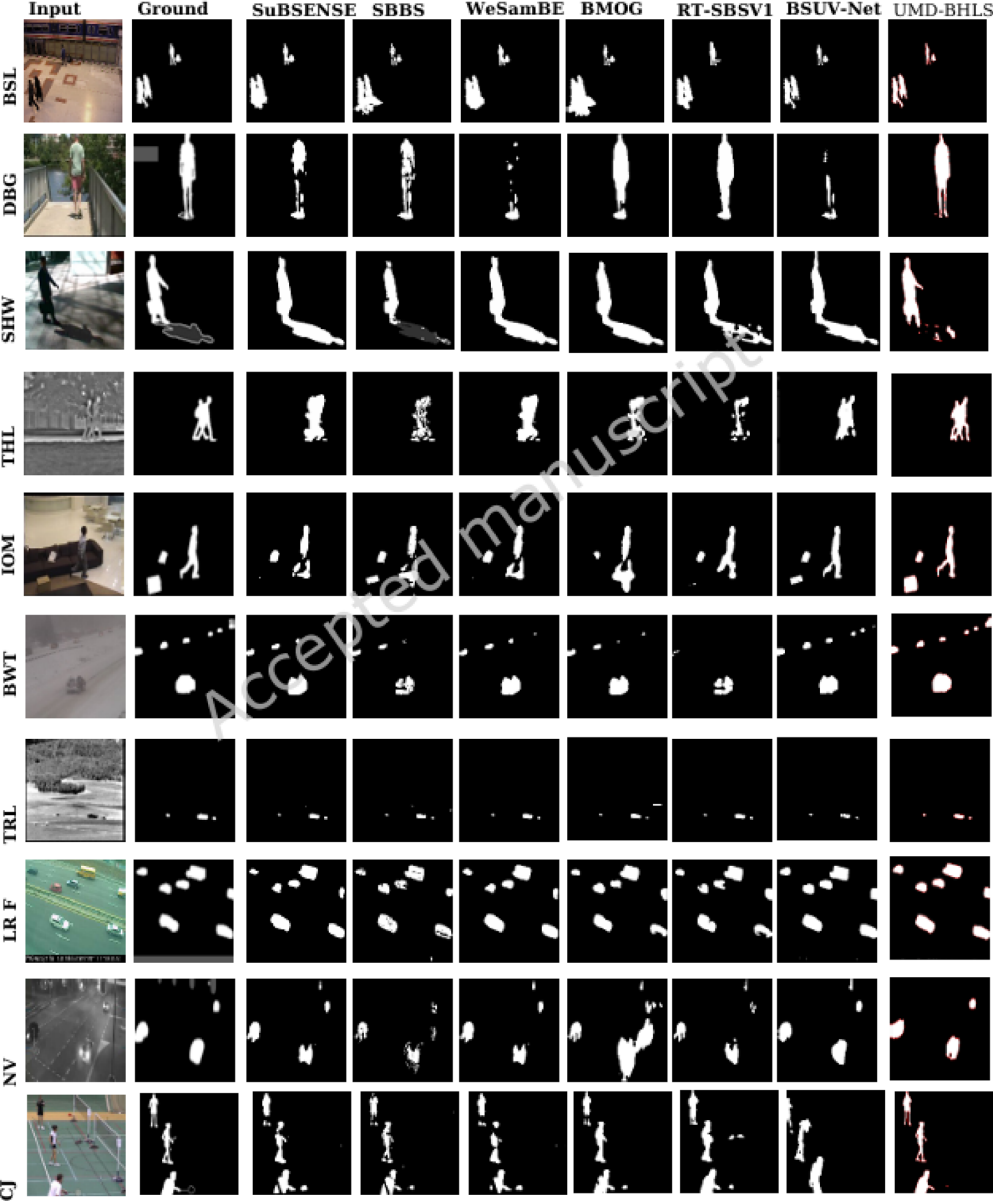
Qualitative comparison of segmentation results on sample frames from ten challenging categories of the CDnet-2014 dataset^62^. Columns display, from left to right: Input Image, Ground Truth, and the outputs of SuBSENSE, SBBS, WeSamBE, BMOG, RT-SBS, BSUV-Net 2.0, and the proposed UMD-BHLS method.

To complement the quantitative analysis and demonstrate the practical robustness of our approach, a detailed qualitative comparison has been applied on the previous nine challenging video scenes, as illustrated in Fig. 7. Column one contains the input frames, column two provides the corresponding ground truth, while column ten illustrates the segmentation results of our approach. Column s 2 to 9 present the results of the SuBSENSE^22^, SBBS^20^, WeSamBE^23^, BMOG^19^, RT-SBS^25^, and BSUV-Net 2.0^15^ methods, respectively. Lines 1 to 10 show the selected sequences: PETS2006 (BSL), Overpass (DBA), Backdoor (SHW), Park (THL), Sofa (IOM), Blizzard (BWT), Turbulence0 (TRL), Turnpik_0.5fps (LFR), and StreetCornerAtNight (NV), peopleInShade (CJ). These scenarios include a large range of visual challenges like low illumination, weather disturbances, shadow cast, camera jitter, and low-frame-rate features. In all scenarios, the proposed method produces cleaner masks and more accurate boundaries, and improves resilience to complex indoor and outdoor scenarios. In the BSL sequence, which comprises moderate crowd and an abandonment object, most baseline methods tend to misclassify shadow regions, struggle to separate objects and produce inflating segmentation masks. BSUV-Net 2.0^14^ exhibit minor ghosting and edge noise. Our approach provides improvements over current best-performing segmentation models, by effectively discriminating moving object boundaries with well-delineated foreground contours, where the FAST-ICA process removes redundant background patterns, while the global energy term enhances consistent foreground extraction. In the DBA sequence, which is characterized by an unsteady background (e.g., waving trees, water ripples), our approach outperforms those of state-of-the-arts methods, notably WeSamBE^23^ and BSUV-Net 2.0^14^. It effectively distinguishes moving objects, filters out background motion, and delivers a clean foreground mask closely aligned with the ground truth, whereas other methods demonstrate slight over-segmentation due to global intensity adaptation and suffer from background leakage or fragmented foregrounds. The local energy function within our model adapts dynamically to spatial variations and background movements, while the FAST-ICA effectively removes repetitive dependent background patterns. The Shadow sequence tests the model’s robustness to dynamic lighting variation and shadow. Here, the proposed hybrid model incorporates spatial and contextual features to effectively discriminate the foreground object in such environmental conditions, where the global energy term distinguishes the right object region, while the local region term helps in preserving object shapes and partially reducing shadows. Other methods like SBBS^20^, WeSamBE^23^, and BSUV-Net 2.0^14^ frequently misclassify shadows as foreground regions, resulting in inflated or distorted segmentations. For thermal imagery that is characterized by low contrast and ambiguous boundaries, most methods deliver fragmented or incomplete masks. The BSUV-Net 2.0 method achieves moderate performance compared to our approach, which handles the thermal data robustly.

The Fast-ICA isolates effectively thermal anomalies, and the local energy term fits contour evolution around weak gradients and deals well with intensity homogeneity. This leads to deliver full object silhouettes that closely resemble the ground truth. When treating IOM sequence, foreground objects may become temporarily stationary as shown, in the Sofa sequence, most baseline methods including SBBS^20^, WeSamBE^23^, and BMOG^19^ erroneously absorb static foreground into the background model and add ghost effect. In contrast, BSUV-Net 2.0^14^, and our method identify correctly objects across intermittent motion cycles and maintain spatial and temporal continuity without adding a ghost effect. Our hybrid model ensures temporal consistency by using ICA decomposition memory, and the level set model preserves object boundaries across frame.

This combined approach allows persistent tracking, handling both stationary and intermittently visible objects. In highly noisy environments, particularly fog and snow, as shown in the BWT sequence, our method achieves the best performance in bad weather conditions, reliably defines object boundaries, and delivers segmentation masks nearly similar to the ground truth, followed closely by BSUV-Net 2.0^14^, SBBS^20^, and RT-SBS^25^ fail to preserve object structure and misclassify moving objects. Atmospheric distortions in the TRL sequence present another challenging test, where most methods show high instability and misrepresent moving objects. SuBSENSE^22^, SBBS^20^, and BMOG^19^ produce significant fragmentation or shape warping. BSUV-Net 2.0^14^ provides partial stabilization but still suffers from fine motion patterns. The proposed method handles warping effectively and motion distortion, maintains consistency, and preserves object shapes and edges. While FAST-ICA extracts statistically significant motion features, adaptive local energy terms adapt dynamically to distorted boundaries. The NV sequence in night-time, characterized by poor illumination and strong artificial light sources, most methods misclassify headlight glare and ambient reflections. SuBSENSE^22^, BMOG^19^, and SBBS^20^ produce noisy foreground maps and frequent misclassification of background pixels. WeSamBE^23^ and RT-SBS^25^ fail to extract meaningful contours in the dark. BSUV-Net 2.0^14^ performs better but still lacks detail. The proposed model substantiates resilience by accurately delineating foreground motion from lighting noise, resulting in a clean and accurate mask. Finally, a distinct challenging category is camera jitter, where slight camera shift or mechanical instability causes background displacement across frames. This leads to many methods, such as WeSamBE^23^ and RT-SBS^25^, to falsely interpret background motion as foreground, producing noisy and fragmented masks. Where the proposed method deals effectively with camera shaking. By modeling global spatial structures through ICA and refining edges locally, it accurately discriminates between actual object motion and global frameshifts. The resulting segmentation is free of spurious motion artifacts and closely matches the ground truth. Overall, our approach exhibits superior qualitative performances in extreme and challenging conditions, including thermal domains, low illumination, and dynamic environments. It delivers clean and accurate segmentations, whereas other methods fail due to environmental complexities or modeling limitations.

### Real-world application: abandoned object detection in crowded surveillance scenes

To further validate the practical effectiveness and operational robustness of the proposed UMD-BHLS framework beyond standard benchmark evaluations, this section presents an application-oriented analysis using the PETS2006 surveillance sequence, a canonical scenario widely adopted for assessing abandoned object detection in crowded public environments. This scenario embodies several critical real-world challenges, including high pedestrian density, frequent and prolonged occlusions, complex object interactions, illumination fluctuations, and most importantly the need to reliably distinguish temporarily static foreground objects from the true background.

**Fig. 8:**
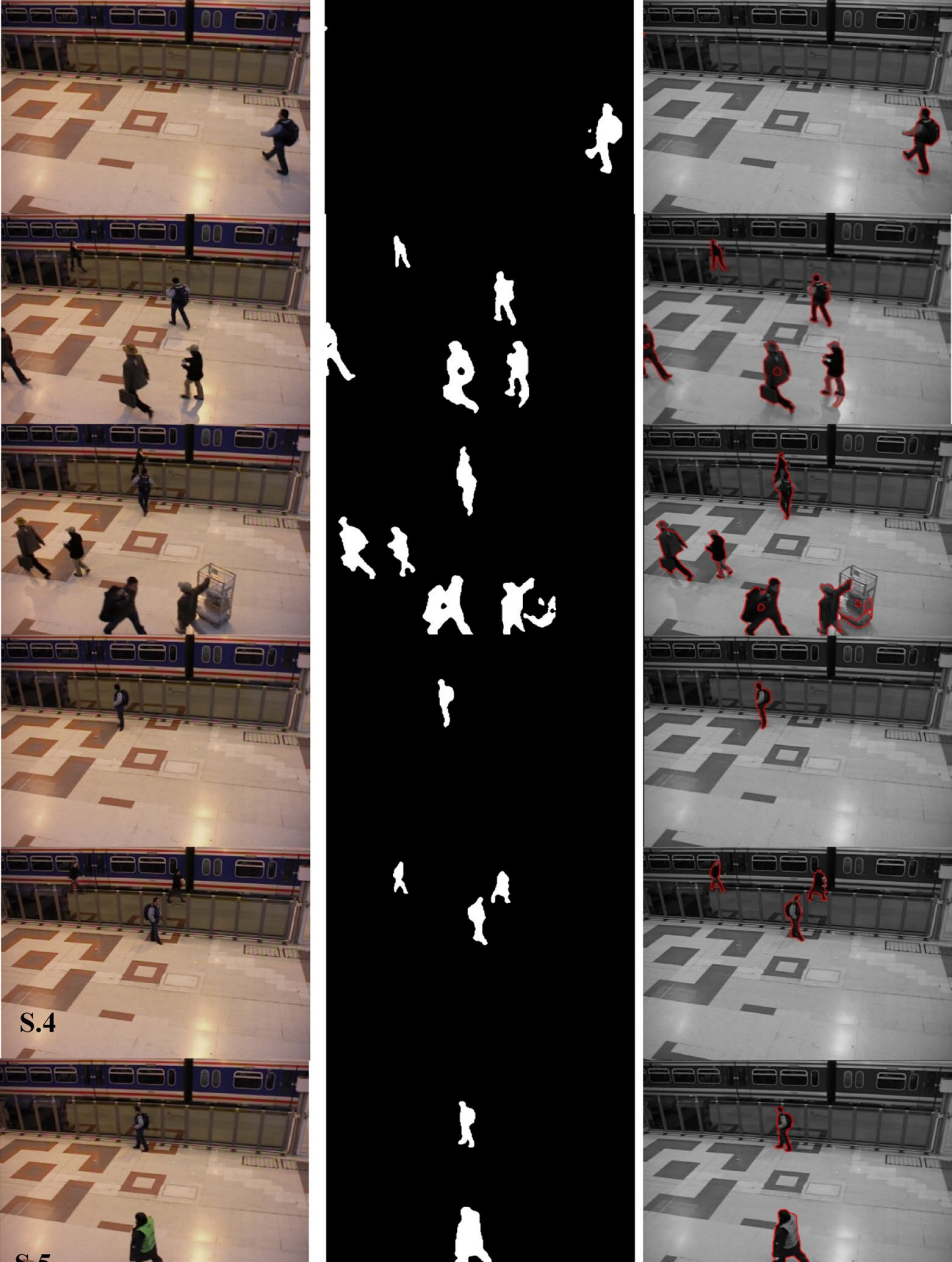

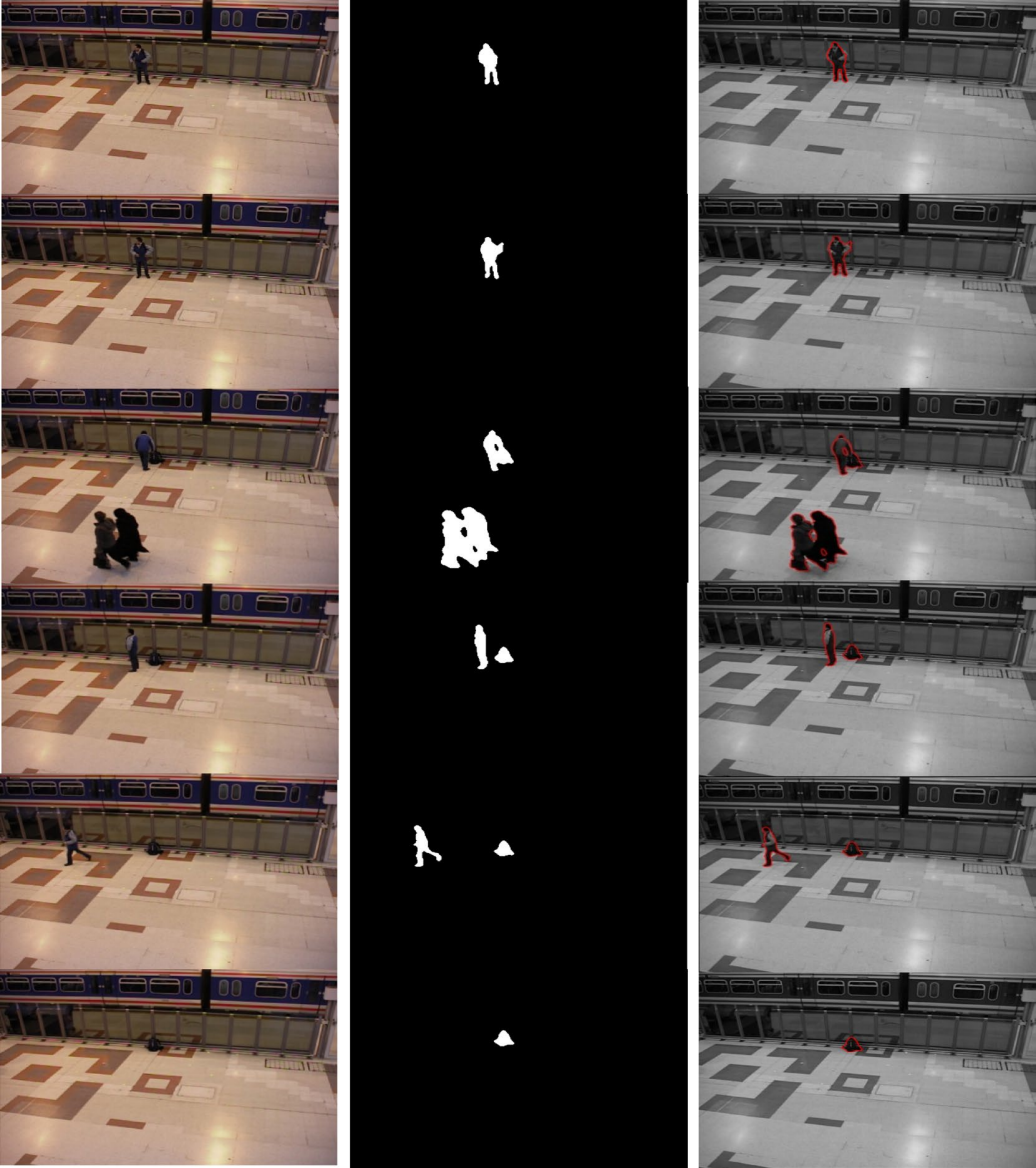
Scenario of abandoned object detection in the station hall “PETS2006“^62^. Col 1: Input images. Col 2: Initial motion masks from the Fast-ICA stage. Col 3: Final segmentation results after adaptive hybrid level-set.

Figure 8 illustrates a representative 12-frame subsequence from the PETS2006 station hall sequence. For each frame, three visual outputs are shown: the original input image, the initial motion mask produced by the Fast-ICA stage, and the final refined segmentation obtained after adaptive hybrid level-set evolution.

This application rigorously tests the two-stage architecture of UMD-BHLS. The Fast-ICA statistical decomposition provides a high-recall initialization by isolating moving entities despite crowding and shadow interference. Subsequently, the adaptive hybrid level-set model refines these coarse detections into spatially coherent and topologically consistent object masks. In this refinement stage, the global Chan-Vese term ensures stable contour evolution and preserves region consistency in homogeneous background areas such as floors and walls, while the local contrast-driven term enhances boundary localization around pedestrians and small objects under occlusions and weak contrast conditions. The complementary interaction between these global and local forces enables accurate segmentation even in complex crowded scenes.

This experiment demonstrates that UMD-BHLS delivers stable and reliable foreground extraction in realistic surveillance scenarios, providing an effective perceptual basis for higher-level security tasks such as abandoned object detection.

#### Frame-by-frame analysis

The sequence narrates a critical event: a person (Subject A) deposits a backpack and leaves the scene. The performance of UMD-BHLS is analyzed at key stages:

- Frames S.1–S.4 (Crowd Ingress and Occlusion Handling): Multiple pedestrians enter the scene, frequently overlapping and partially occluded by structural elements. The Fast-ICA stage successfully isolates all moving subjects, including those with fragmented visibility. The subsequent level-set refinement preserves distinct object contours despite close proximity and topology changes, highlighting the advantage of implicit contour representation and adaptive regularization.
- Frames S.5–S.8 (Subject Stationarity and Foreground Persistence): Subject A becomes temporarily stationary. Conventional background subtraction methods typically begin absorbing such static objects into the background, resulting in ghosting artifacts or gradual disappearance. In contrast, UMD-BHLS maintains the subject as foreground. This behavior arises from the statistical independence modeling of Fast-ICA, which prevents premature background assimilation, combined with the temporal coherence of the evolving level-set function.

- Frames S.9–S.10 (Object Deposition): The backpack is placed on the ground, forming a new, non-human foreground object with weak contrast against the floor. The adaptive local energy term plays a crucial role at this stage, sensitively capturing subtle gradient variations between the object, the subject, and the surrounding background. This enables accurate separation and prevents merging artifacts.
- Frames S.11–S.12 (Abandonment and Persistent Detection): After Subject A leaves the scene, the backpack remains isolated. This constitutes the definitive test for abandoned object detection. UMD-BHLS continues to segment the backpack as foreground without decay. This persistence is ensured by the continued statistical distinction from the background and by the adaptive stabilization term $\:{\eta\:}_{t}R\left(\phi\:\right)$, which prevents contour diffusion or collapse around static foreground objects. The final segmentation (S.12) yields a clean, stable object mask suitable for triggering higher-level security alerts.

This application scenario is included as an illustrative real-world surveillance scenario to demonstrate the practical behavior of the proposed UMD-BHLS framework in the context of abandoned object detection. This analysis shows how the two-stage architecture combining Fast-ICA statistical separation with adaptive hybrid level-set refinement responds to crowded scenes, occlusions, and temporarily static objects. The results validate the framework’s capacity to maintain persistent detection of a deposited object without ghosting or background absorption, demonstrating its practical viability as a perceptual foundation for higher-level surveillance tasks under realistic conditions.

### Failure cases and limitations analysis

**Fig. 9:**
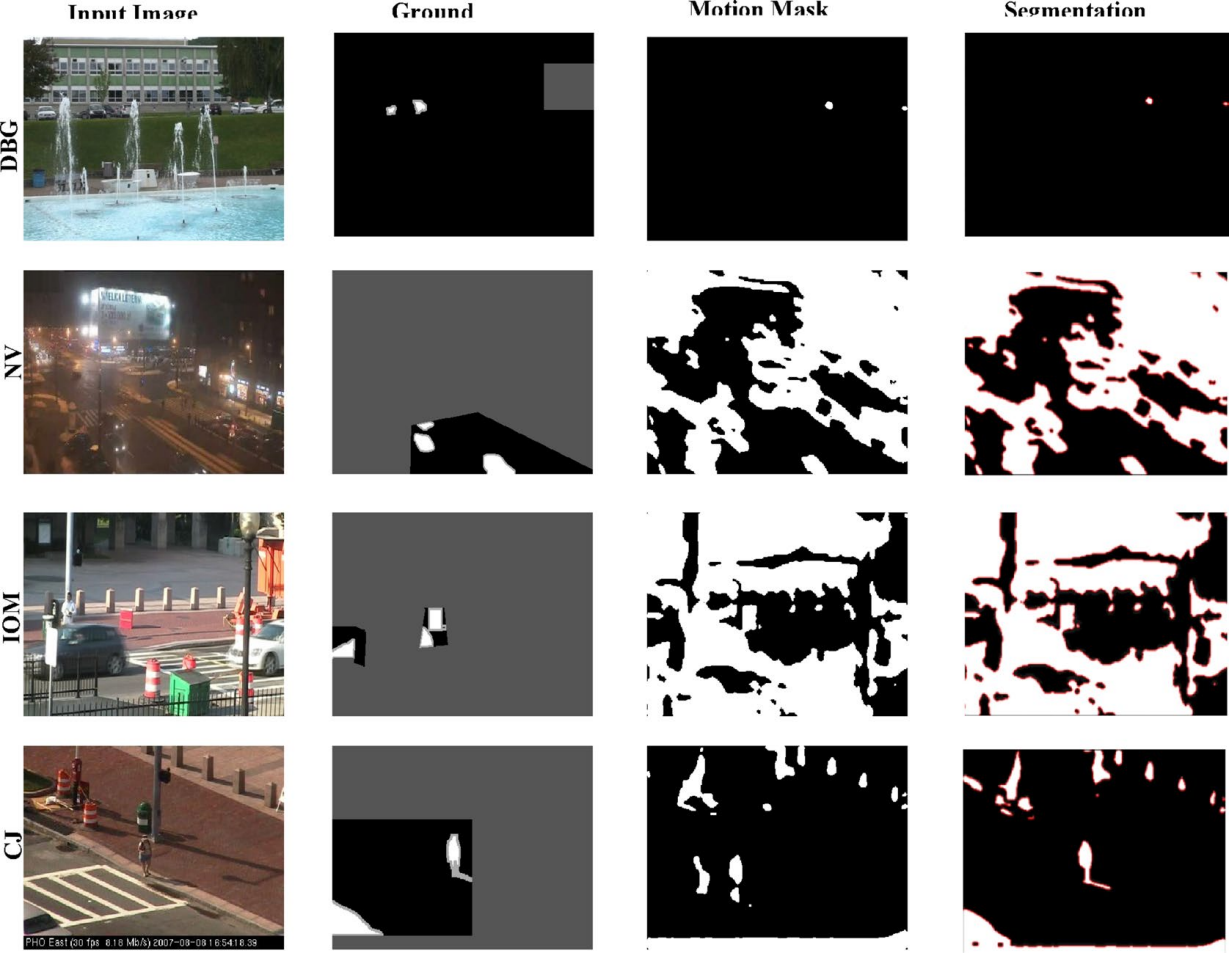
Representative failure cases of the proposed UMD-BHLS method on challenging CDNet-2014 sequences^62^: Dynamic Background (DB), Night Video (NV), Intermittent Object Motion (IOM), and Camera Jitter (CJ). From left to right: input image, ground truth, initial motion mask (Fast-ICA), and final segmentation result.

Although the proposed UMD-BHLS framework demonstrates strong robustness across most CDNet-2014 categories, several challenging scenarios may still affect segmentation accuracy. Figure 9 presents representative failure cases illustrating the main limitations of motion-based foreground detection under complex environmental conditions.

In the Fountain01 (DBG) sequence, continuous water motion generates strong intensity fluctuations that resemble foreground activity, leading to occasional false detections during the motion separation stage. While the hybrid level-set refinement reduces part of these artifacts, some residual errors remain due to the similarity between dynamic background motion and true object motion.

The Busyboulevard (NV) sequence highlights the effect of poor illumination and sensor noise. Under low-light conditions, the contrast between foreground objects and the background decreases, which weakens the reliability of statistical motion separation and may produce fragmented foreground masks.

In the Tramstop (IOM) sequence, objects that remain stationary for extended periods may gradually merge with the background model. When motion resumes, detection may appear delayed or partially fragmented, affecting the subsequent contour refinement.

Finally, the Sidewalk (CJ) sequence illustrates the impact of small camera vibrations, where slight global motion may be misinterpreted as foreground activity. Although spatial regularization in the hybrid level-set model alleviates some of these effects, minor false positives may persist. Despite these challenges, the proposed framework remains robust across most surveillance scenarios. Future work will focus on incorporating camera motion compensation, improved temporal modeling, and enhanced dynamic background suppression to further strengthen performance under extreme conditions.

## Conclusion

In this work, we propose a new and robust method for automated visual motion detection and segmentation, which is meticulously developed to satisfy the demanding requirements of intelligent video surveillance systems operating in both indoor and outdoor environments. The proposed method adopts a dual-core structure: motion detection based on an improved FAST-ICA technique for statistical foreground-background decomposition, then segmentation refinement with hybrid level set model, which optimally evolves contours using both global energy regularity and local spatial features. This hybridization allows the system to capture both large-scale intensity homogeneity and fine local structures, ensuring accurate boundary localization and reducing leakage and over-segmentation problems, even in complex background dynamics, environmental noise, temporal discontinuities, and low-visibility conditions. In addition, our approach maintains the smoothness and the regularization of the evolving level set function thanks to the stabilization term, which plays a substantial role in enhancing the precision, robustness, and stability of the segmentation process. It also incorporates self-regulating stopping criterion based on energy convergence, further enhancing segmentation stability and preventing unnecessary computational overhead. Through extensive quantitative and qualitative comparisons across challenging CDNet-2014 sequences, our method achieves superior performance in all categories. Compared to classical and even advanced deep learning methods, the proposed approach offers a compelling compromise between accuracy, adaptability, and computational efficiency, ensuring its suitability for real-world deployment in surveillance, autonomous systems, and environmental monitoring.

Despite its strong performance, UMD-BHLS has some limitations. It is mainly designed for static camera surveillance and does not explicitly handle PTZ scenarios with significant global background motion. In addition, limitations of the proposed framework arise in scenes involving highly dynamic backgrounds, severe illumination degradation, intermittently static objects, and camera instability. Future improvements could focus on incorporating camera motion compensation, enhanced temporal modeling, and stronger dynamic background suppression to further improve robustness in such conditions.

## Data Availability

Availability of data and materials Datasets used and/or analyzed during the current study are available from the corresponding author upon reasonable request.
